# Comprehensive Transcriptome and Proteome Analyses Reveal the Drought Responsive Gene Network in Potato Roots

**DOI:** 10.3390/plants13111530

**Published:** 2024-05-31

**Authors:** Tianyuan Qin, Yihao Wang, Zhuanfang Pu, Ningfan Shi, Richard Dormatey, Huiqiong Wang, Chao Sun

**Affiliations:** 1 State Key Laboratory of Aridland Crop Science, College of Agronomy, Gansu Agricultural University, Lanzhou 730070, China; qinty@st.gsau.edu.cn (T.Q.); wangyh@st.gsau.edu.cn (Y.W.); puzf@st.gsau.edu.cn (Z.P.); shinf@st.gsau.edu.cn (N.S.); wanghq@st.gsau.edu.cn (H.W.); 2 CSIR—Crops Research Institute, P.O. Box 3785, Kumasi 00233, Ghana; rmddormatey@gmail.com

**Keywords:** potato, drought stress, transcriptome, proteome, gene network

## Abstract

The root system plays a decisive role in the growth and development of plants. The water requirement of a root system depends strongly on the plant species. Potatoes are an important food and vegetable crop grown worldwide, especially under irrigation in arid and semi-arid regions. However, the expected impact of global warming on potato yields calls for an investigation of genes related to root development and drought resistance signaling pathways in potatoes. In this study, we investigated the molecular mechanisms of different drought-tolerant potato root systems in response to drought stress under controlled water conditions, using potato as a model. We analyzed the transcriptome and proteome of the drought-sensitive potato cultivar Atlantic (Atl) and the drought-tolerant cultivar Qingshu 9 (Q9) under normal irrigation (CK) and weekly drought stress (D). The results showed that a total of 14,113 differentially expressed genes (DEGs) and 5596 differentially expressed proteins (DEPs) were identified in the cultivars. A heat map analysis of DEGs and DEPs showed that the same genes and proteins in Atl and Q9 exhibited different expression patterns under drought stress. Weighted gene correlation network analysis (WGCNA) showed that in Atl, Gene Ontology (GO) terms and Kyoto Encyclopedia of Genes and Genomes (KEGG)-enriched pathways were related to pyruvate metabolism and glycolysis, as well as cellular signaling and ion transmembrane transporter protein activity. However, GO terms and KEGG-enriched pathways related to phytohormone signaling and the tricarboxylic acid cycle were predominantly enriched in Q9. The present study provides a unique genetic resource to effectively explore the functional genes and uncover the molecular regulatory mechanism of the potato root system in response to drought stress.

## 1. Introduction

Potato (*Solanum tuberosum* L.) is an important tuber crop and one of the four most productive crops worldwide. Global potato production increased from 350 billion kilograms to 368 million metric tons, with current production set to reach 375 million metric tons in 2016, 2018, and 2022, respectively [1]. China is one of the largest potato producers globally, with the main potato-growing areas located in arid and semi-arid regions with low rainfall, such as Northwest China, Western Inner Mongolia, and Northeast China [2]. However, potato cultivation in China faces major challenges due to drought and limited water resources. Consequently, high potato yields in these regions depend on extensive irrigation, which poses a number of environmental problems [3,4]. In addition, prolonged or seasonal drought has a significant impact on plant growth, tuber yield, and potato marketing [5,6]. Especially during the growth phase of the tubers, severe drought stress occurs, which can lead to significant yield losses or crop failure [7,8]. Therefore, thorough research into the drought tolerance of potatoes is imperative.

Roots play a pivotal role throughout a plant’s growth cycle because they form com plex root system structures through growth and branching to fulfill the plant’s rooting function in the soil and absorb water and nutrients [9,10]. Certain crops, such as rice. and the model-plant *Arabidopsis thaliana* (L.) Heynh. exhibit dominant root phenotypes characterized by deep rooting and gravity-oriented growth, contributing to their drought adaptation. These root phenotypes help plants to efficiently absorb water resources from deeper soil layers, thus ensuring normal plant biological activities [11,12]. In addition, growth in the direction of gravity is an important characteristic of the root type when adapting to drought. When plants sense drought stress, the root system is able to grow gravitropically, i.e., it grows downward to improve vertical extension in the soil. This increases the contact area between the root system and soil and increases the water and nutrient uptake efficiency. Recent findings of genes involved in deeper rooting have contributed significantly to our comprehensive understanding of the mechanisms underlying plant adaptation to drought and helped us to facilitate appropriate root modulation studies to improve drought resistance in crops [13]. Researchers identified a gene that provides deeper rooting [13], and others have found that phosphorylation enhances SWEET protein oligomerization and their sucrose transport activity. This promotes long-distance transport from the aerial parts to the root system, promoting root system growth under drought conditions and improving the root-to-crown ratio and drought resistance in plants [14]. 

In response to insufficient water availability in the soil, plants modify their root architecture to increase water uptake capacity. Morphological changes, including an increase in root hair density, apical region elongation, and increased hydraulic conductivity in xylem canals, contribute to this increased water uptake capacity. Deep rooting and smaller branching angles in root architecture have been shown to improve a plant’s ability to absorb water from deeper soil layers. This root shape is a strategy used by plants to adapt to dry environments to ensure the stability and durability of water uptake [15]. In addition, in model plants such as *Arabidopsis thaliana*, the HCR and XND1 genes have been found to play important roles in regulating water conductivity in the root system, and mutations in the HCR1 gene lead to a decrease in lignin in the root cell wall, altering the water permeability and water conductivity of the cell wall; xND1 gene expression is closely related to the corking process, and down-regulation of XND1 gene expression in a mutant led to an increase in water conductivity in the root system [16]. In addition, xylem canals play important roles in plants by modulating the diameter of the xylem canal passages and chambers to control water and nutrient transport rates and the conduction of photosynthetically assimilated products to the roots through the phloem and maintaining proper osmotic pressure in tissues. Efficient water uptake and distribution allow plants to cope effectively with drought conditions [16].

In general, several traits can be used to screen plant roots for drought tolerance, including the ability of plants to germinate in soils with different osmotic potentials, the depth, width, and strength of the root system in the soil, the status of starch hydrolysis in the root crown under drought conditions, and the antioxidant enzyme activity in the root system and root vigor (RV) [17,18,19]. Moreover, morphological indicators such as root growth and yield indicators can also be used to assess the drought tolerance of crops [20,21]. Therefore, a comprehensive evaluation combining the above indicators can more accurately reflect the actual drought resistance of crops. Since the root system is the main organ for plant water uptake, a comprehensive understanding of the genes related to root growth and development is required. The emergence of genotypes and potato varieties that differ in their drought tolerance provides an excellent opportunity to uncover the genetic factors involved in the root system’s response to drought stress in different drought-tolerant varieties. Consequently, in this study, the transcriptomes and proteomes of two potato cultivars showing significant differences in drought tolerance were analyzed at different stages in the response to drought stress by applying RNA-seq (RNA sequencing) and DIA (data-independent acquisition) techniques. These analyzed data revealed the transcriptome and transcriptional network dynamics associated with the root system’s response to drought stress and identified key genes involved in the root response to drought stress in different drought-tolerant potato cultivars. In addition, genes and co-expressed gene modules that are predominantly or specifically expressed at different stages of the root response to drought stress or within a particular cultivar were identified

## 2. Results

### 2.1. Results of the Sequencing of the Entire Transcriptome and Proteome

Correlation analysis revealed correlation coefficients between samples ranging from 0.98 to 1 (Supplementary Figure S1A), exceeding the Encode program’s recommended value of 0.92 for optimal sampling and testing conditions. A correlation coefficient of 1 indicates higher similarity between samples and a lower number of differentiated genes, indicating reliable sampling and sequencing results for all samples tested. Whole transcriptome sequencing over three generations yielded 305,015,810 raw reads from 24 samples. Using the bioinformatics software, we obtained a total of 289,335,442 clean reads, all of which had a Q30 value of over 95.48% and a GC content of 52.14% to 56.14%. In addition, the ratio of clean reads to total raw reads was above 91.71% for all reads (Supplementary Table S1). In the proteome, the correlation coefficients between the three replicates of each sample also ranged from 0.98 to 1 (Supplementary Figure S1B), and a total of 76,410 peptides and 6771 proteins were identified from the 24 samples using the DIA-targeted extraction quantification algorithm DIA-NN, which was verified with a false discovery rate (FDR) of 1.

### 2.2. Analysis of Differential Genes and Proteins

**Figure 1:**
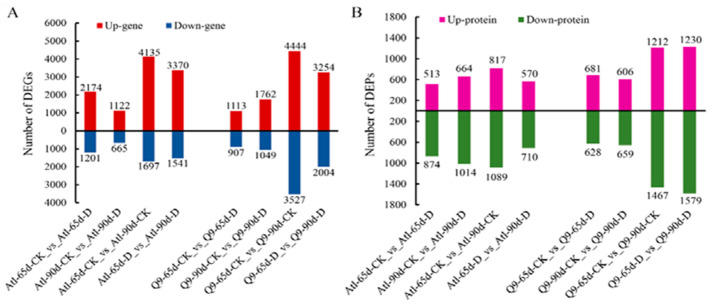
Pairwise comparative analysis of DEGs (**A**) and DEPs (**B**) in Atl and Q9 roots under drought stress.

The results of the samples (Atl and Q9) at different time points compared to their respective control (CK) for significantly up- and down-regulated differentially expressed genes (DEGs) and differentially expressed proteins (DEPs) are shown in Figure 1A,B. The results showed that after 65 days of drought stress when Atl was used as a control (CK), 2174 genes and 513 proteins were upregulated, while 1201 genes and 874 proteins were downregulated. After 90 days of stress, 1122 genes and 664 proteins were upregulated, while 665 genes and 1014 proteins were downregulated. In the case of Q9, after 65 days of drought stress, 1113 genes and 681 proteins were upregulated, while 907 genes and 628 proteins were downregulated. After 90 days of stress, 129 genes and 606 proteins were upregulated, while 1089 genes and 659 proteins were downregulated (Figure 1A,B).

**Figure 2:**
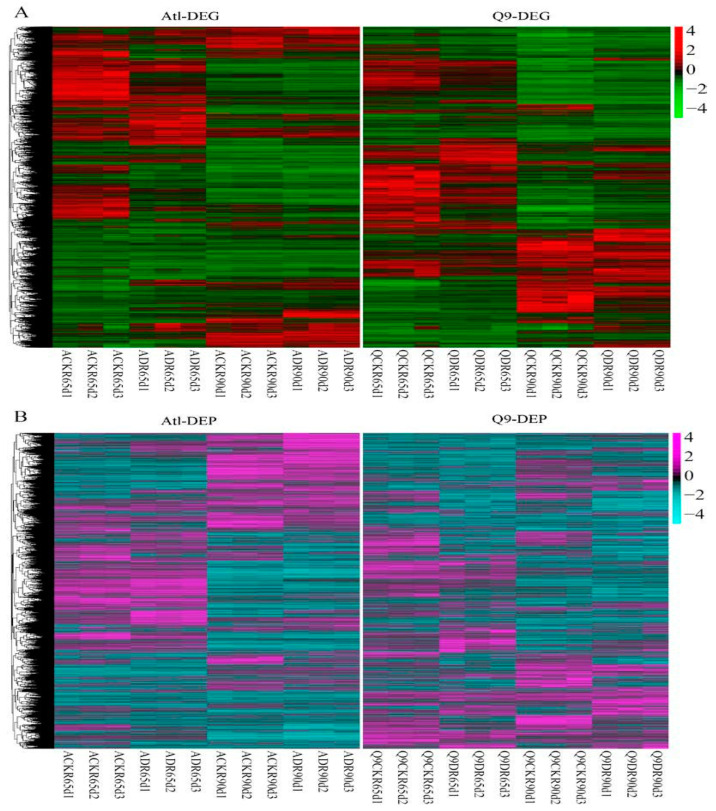
Heatmap of DEG (**A**) and DEP (**B**) expression in Atl and Q9 roots under drought stress. The order of the genes from top to bottom is the same on the left and right panels of both figures.

We also obtained a total of 14,113 DEGs and 5596 DEPs after de-duplicating all DEGs and DEPs analyzed above. Analysis of the expression heat maps of these DEGs and DEPs revealed that the same genes and proteins showed different expression patterns in the Atl and Q9 cultivars when exposed to drought stress conditions (Figure 2A,B).

**Figure 3:**
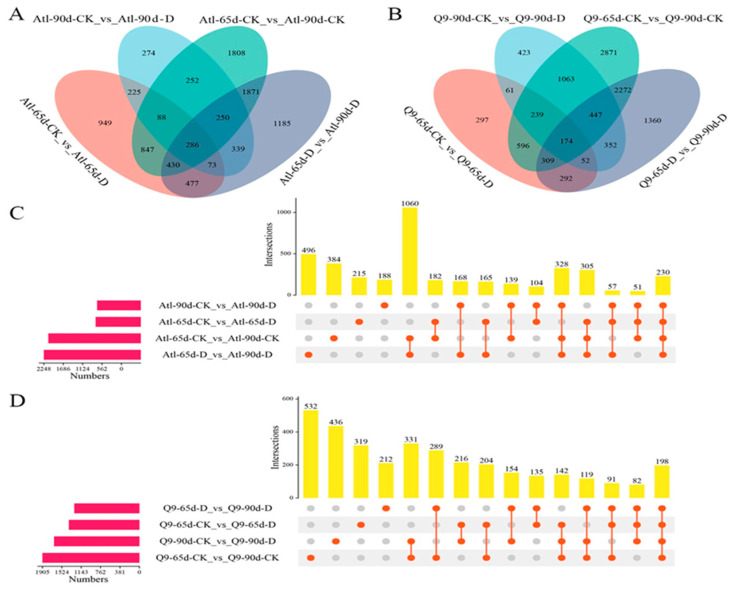
Venn analysis of DEGs (**A**,**B**) and DEPs (**C**,**D**) in Atl and Q9 roots under drought stress. (**A**,**B**) represent the number of DEGs in the Atl and Q9 roots; (**C**,**D**) represent the number of DEPs in the Atl and Q9 roots. The pink bar represents the total number of proteins expressed in the sample; the yellow bar represents the number of proteins expressed in the sample in the Atl and Q9 roots; the pink bar represents the total number of proteins expressed in the sample; the yellow bar represents the number of DEPs in the sample corresponds to the orange dot.

The results of the expression patterns of different genes and proteins using Venn diagram [22] showed that different genes (Figure 3A) and 230 different proteins (Figure 3C) were expressed in Atl in all four combinations of watering treatment or developmental stage: Atl-65d-CK_vs_Atl-65d-D, Atl-90d CK_vs_Atl-90d-D, Atl-65d-CK_vs_Atl-90d-CK, and Atl-65d-D_vs_Atl-90d-D. In addition, there were 225 common differential genes (Figure 3A) and 104 common differential proteins (Figure 3C) that were co-expressed in the Atl-65d-CK_vs_Atl-65d-D and Atl-90d-155 CK_vs_Atl-90d-D combinations. In addition, the combinations that compared the development stage at each treatment, Atl-65d-CK_vs_Atl-90d-156 CK and Atl-65d-D_vs_Atl-90d-D had a combined total of 1871 different genes (Figure 3A) and 1060 different proteins (Figure 3C). In the Q9 variety, the four combinations of watering treatment or developmental stage, Q9-65d-CK_vs_Q9-65d-D, Q9-90d-CK_vs_Q9-90d-D, Q9-65d -CK_vs_Q9-90d-CK and Q9-65d-D_vs_Q9-90d-D had 174 different genes (Figure 3B) and 198 different proteins (Figure 3D). Furthermore, 61 different genes (Figure 3B) and 216 different proteins (Figure 3D) were 161 co-expressed in the two combinations, which compared watering treatments at each development stage, Q9-65d-CK_vs_Q9-65d-D, and Q9-90d-CK_vs_Q9-162 90d-D. In addition, the combinations that compared the development stage at each treatment, Q9-65d-CK_vs_Q9-90d-CK and Q9-65d-D_vs_Q9-163 90d-D shared 2272 different genes (Figure 3B) and 289 different proteins (Figure 3D).

**Figure 4:**
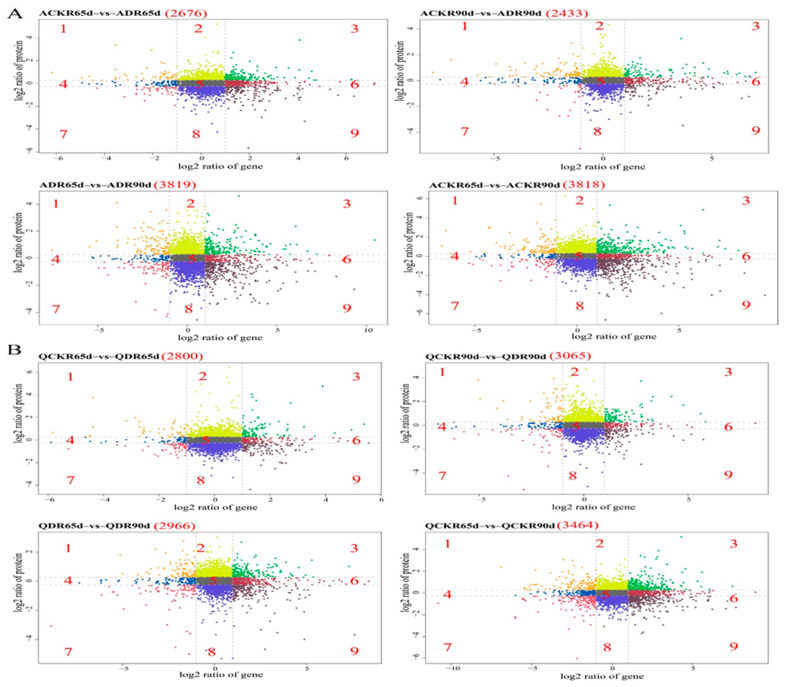
Association analysis of DEGs and DEPs in Atl (**A**) and Q9 (**B**) roots (R) under control or drought stress at 65 or 90 d. In red parenthesis are the number of genes identified in the nine quadrants.

A correlation analysis of the differences in the expression of DEGs and DEPs using a nine-quadrant plot is shown in Figure 4. Quadrants 1, 4, and 7 identified the mRNAs that are negatively correlated with the corresponding DEP patterns; quadrants 2 and 8 indicate the proteins that are differentially expressed without changes in the corresponding mRNAs; quadrants 3, 6, and 9 identified the mRNAs that are positively correlated with the corresponding DEP patterns; and quadrant 5 indicated those in which neither the co-expressed mRNAs nor the proteins differed in expression. For example, 735, 731, and 605 genes in quadrants 1, 4, and 7, respectively, showed negative correlations between mRNA and protein expression, while 1251, 1870, and 1874 genes in quadrants 3, 6, and 9, respectively, showed positive correlations between mRNA and DEPs.corresponding DEP patterns and quadrant 5 indicated those for which neither the co-expressed mRNAs nor the proteins differed in expression. 

#### 2.2.1. Heatmap of the Association between Modules and Traits in Gene Co-Expression Networks

The genes were recombined with the modules based on a correlation threshold of 75%. Integration was performed using trait matrices associated with root length (Length), relative root water content (RWC), root crown ratio (RCR), yield, root volume (RV), proline (Pro), malondialdehyde (MDA), peroxidase (POD), catalase (CAT), gibberellin (GA), indoleacetic acid (IAA), salicylic acid (SA), abscisic acid (ABA), and cytokinin (CTK) content. In Atl, 13 modular heatmaps were obtained, while Q9 yielded 16 modular heatmaps (Supplementary Figure S5). Each trait had modules significantly associated with it. The yellow module in Atl showed significant positive correlations with length, RCR, MDA, POD, CAT, GA, and CTK. The turquoise module showed a significant negative correlation with length, RSR, MDA, POD, CAT, GA, and CTK, while the green module also showed a positive correlation with yield. In Q9, the yellow module showed a significant positive correlation with length, RWC, RSR, MDA, POD, CAT, RV, SA, CTK, and yield. While the turquoise module was significantly negatively correlated with length, RWC, RSR, MDA, POD, CAT, RV, SA, CTK, and yield. The blue module showed a significant positive correlation with yield.

#### 2.2.2. Characterized Gene Module Eigengenes in Gene Co-Expression Networks

In this study, we investigated the biological functions of modules associated with strongly associated traits using a heat map. Modules were selected using feature vector genes or module eigengenes (MEs) to enable efficient analysis due to the large number of genes within each module. MEs are used to represent the thousands of genes in a module for correlation analysis. Using this method, the interrelationships between gene modules and modules and the correlation with different traits can be elaborated further to filter for the target gene modules. The higher the correlation between MEs, the higher the correlation within the respective modules. Through the correlation analysis of MEs between two and two, it was found that in Atl, the MEs in the yellow and turquoise modules reached 0.79, and the correlation between the MEs in the green and brown modules reached 0.84 (Supplementary Figure S6A). Genes within the yellow module showed a positive correlation with most physiological and biochemical indices and a negative correlation with most hormonal indices. Conversely, the genes in the turquoise and brown modules showed a negative correlation with most physiological and biochemical indices and a positive correlation with most hormonal indices. In addition, the green module also showed a significant positive correlation with the yield trait. Therefore, these four modules were selected as target gene modules for further investigation in Atl. In Q9, the ME in the yellow module showed a correlation of 0.89 with the ME in the turquoise module. The ME in the blue module had a correlation of 0.88 with the ME in the brown module (Supplementary Figure S6B), and the genes in the yellow module were significantly and positively correlated with most phenotypes and physiological and biochemical indices. In contrast, they were negatively correlated with some horizontal indices. The genes in the turquoise and brown modules were significantly negatively correlated with most phenotypes and physiological and biochemical indices, while they were positively correlated with some hormonal indices. Moreover, the blue module was significantly positively correlated with the yield trait. Therefore, these four modules were selected as target gene modules for the subsequent studies in Q9. 

The expression levels of both genes and MEs within these eight modules were analyzed individually. It was found that the genes within a module showed a strong positive correlation in their expression levels. In addition, the ME expression levels showed a significant positive correlation with the overall expression levels in a module, indicating that the MEs of the target modules can adequately represent the overall genes in a module (Supplementary Figure S7).

#### 2.2.3. Gene Ontology Enrichment Analysis of Genes in Candidate Modules in Gene Co-Expression Networks

**Figure 5:**
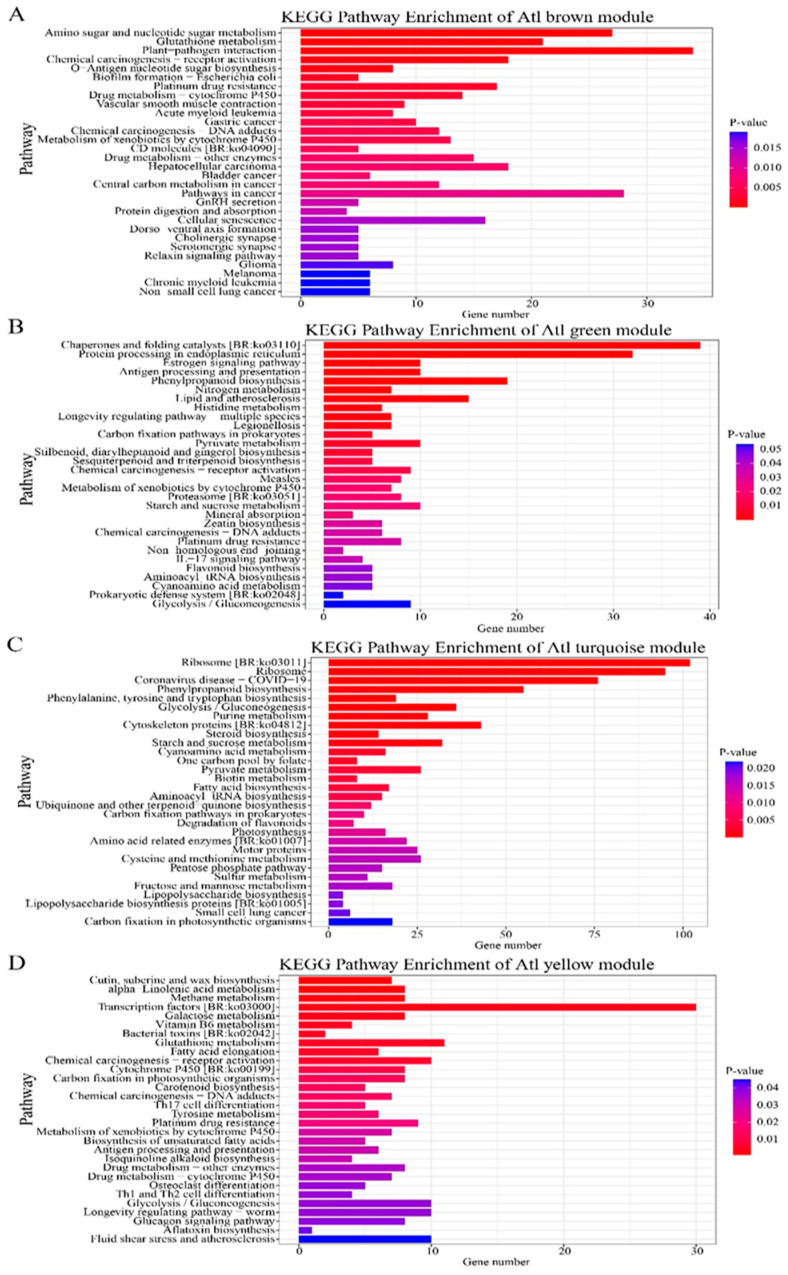

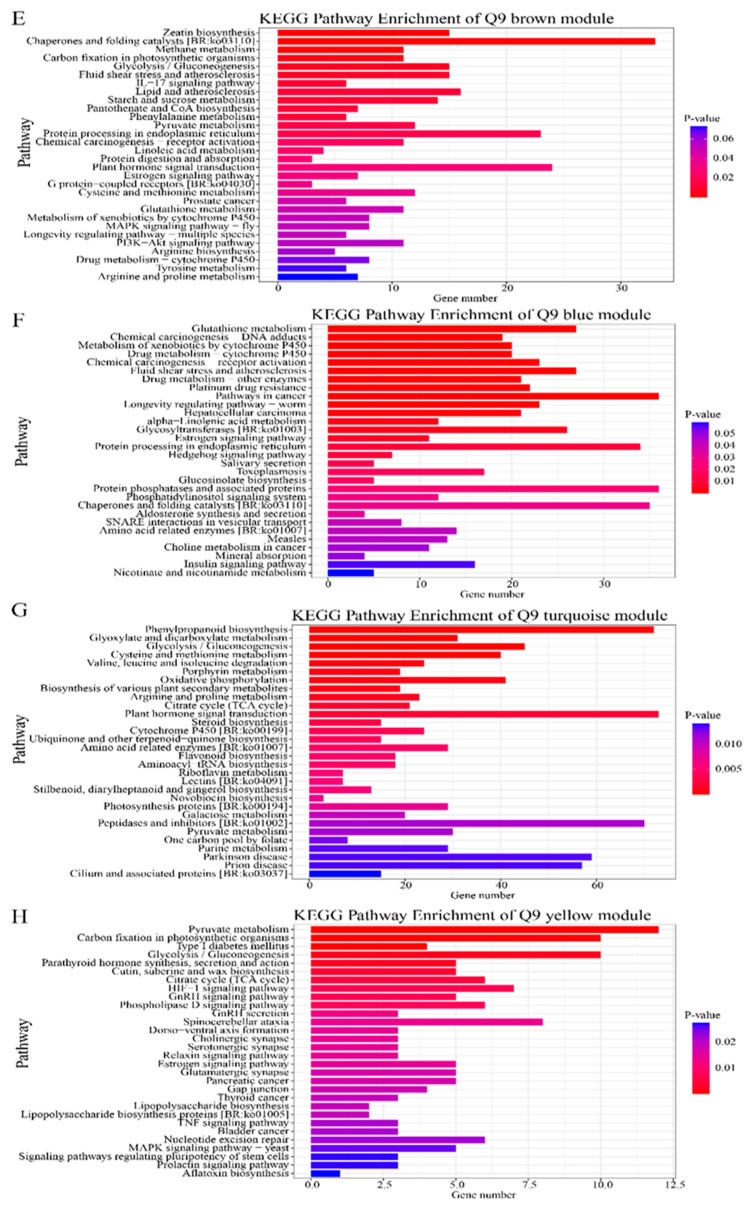
KEGG enrichment of genes in Atl (**A**–**D**) and Q9 (**E**–**H**) candidate modules.

The R package cluster profile was used to analyze the functions of gene ontology in the candidate modules brown, green, yellow, and turquoise in Atl and brown, blue, yellow, and turquoise in Q9. The results showed that all four modules in Atl were enriched with biological processes (BPs) and molecular functions (MFs) (Figure 5A–D). GO terms such as secondary metabolite biosynthetic process, cellular response to DNA damage stimuli, signal transduction regulation, metabolic process of organic hydroxyl compounds, signaling regulation, cellular communication regulation, cellular development, and transmembrane transporter protein activity of inorganic molecular entities were significantly enriched mainly in the brown module. The green module primarily showed enrichment in GO terms related to cellular carbohydrate metabolic processes, precursor metabolites, energy production, response to extracellular stimuli, pigment metabolic processes, α-amino acid metabolic processes, response to SA, and negative regulation of cellular metabolic processes. The yellow module is primarily enriched in GO terms such as cellular response to ABA stimulation, developmental maturation, regulation of cellular communication, cellular carbohydrate metabolic processes, intracellular signal transduction, signal regulation, transferase activity, and identical protein binding. The turquoise module was significantly enriched in GO terms such as organophosphorus biosynthetic processes, pattern determination processes, α-amino acid metabolic processes, secondary metabolite biosynthetic processes, outer organelle membrane, microtubule cytoskeleton, and supramolecular complexes.

In the Q9 genotype, modules were enriched in GO terms for BPs and MFs (Supplementary Figure S8E–H). These included negative regulation of cellular metabolic processes, negative regulation of biosynthetic processes, intracellular protein transport, response to SA, negative regulation of metabolic processes of nitrogen compounds, response to IAA, enzyme regulatory activity, and enzyme binding, which were significantly enriched in the brown module. Response to heat was significantly enriched mainly in the blue module along with secondary metabolite biosynthetic processes, organic hydroxyl compound metabolic processes, signal transduction regulation, cellular communication regulation, ATPase activity, and lyase activity. The yellow module was significantly enriched in the GO terms regulation of cellular communication, cellular response to organic cyclic compounds, external encapsulation and structural organization, carbohydrate biosynthetic processes, cellular carbohydrate metabolic processes, oxidoreductase activity, and ATPase activity. The turquoise module was significantly enriched in cellular carbohydrate metabolic processes, pigment metabolic processes, metabolism of organohydroxy compounds, carbohydrate biosynthetic processes, response to red or far-red light, regulation of cellular communication, negative regulation of cellular metabolic processes, ATP binding, phosphoacetatehydrolase activity, and ATPase activity, and other GO terms. 

#### 2.2.4. Kyoto Encyclopedia of Genes and Genomes Enrichment Analysis of Genes in Candidate Modules in Gene Co-Expression Networks

In the present study, the R package cluster profile was used to analyze KEGG enrichment in the candidate modules examined in Atl and Q9, with a focus on gene function. Based on this analysis, four candidate modules were identified in each genotype. The functional KEGG enrichment in the two potato genotypes showed the four modules as brown, turquoise, green, and yellow, which significantly contained many annotated genes. Each of the color modules is enriched in metabolic pathways, such as “Phenylpropane biosynthesis”, “Nitrogen metabolism”, “Pyruvate metabolism”, “Starch and sucrose metabolism”, “Zeatin biosynthesis”, “Ribosome”, “Phenylalanine–propane biosynthesis”, “Phenylalanine and pyruvic acid metabolism”, “Glutathione metabolism”. Others include “pyruvate metabolism” and “carbon fixation by photosynthetic metabolism”. These metabolic pathways play an important role in the response to abiotic and biotic stress factors in crop production. 

#### 2.2.5. Visualization of the Core Genes in Candidate Modules in Gene Co-Expression Networks

**Figure 6:**
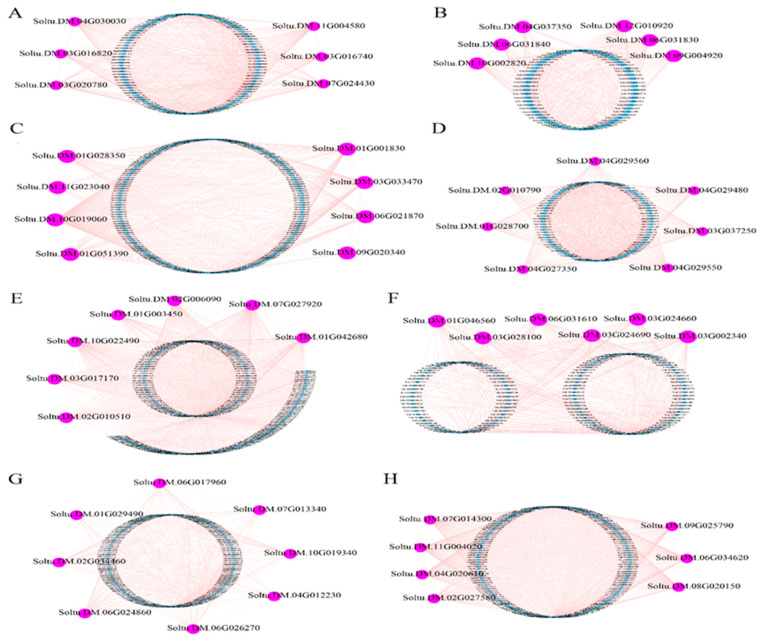
Gene co-expression network and hub gene mining in Atl and Q9 candidate modules. (**A**–**D**) refer to the brown, green, turquoise, and yellow modules in Atl, respectively; (**E**–**H**) refer to the brown, blue, turquoise, and yellow modules in Q9, respectively.

To investigate the functions of the core genes in the eight candidate modules in Atl and Q9, Cytoscape software (version 10.3.1) [23] was used to visualize the gene network in the modules and filter the genes with high connectivity in the modules that were used as core genes in the modules (Figure 6). In this visualization, each node represents a gene, and the connecting lines between the nodes indicate that the genes at the ends of the lines are likely to possess similar biological functions [24,25]. In Atl, six nuclear genes in the brown module, six nuclear genes in the green module, eight nuclear genes in the turquoise module, and seven nuclear genes in the yellow module were selected. In Q9, seven nuclear genes in the brown module, six nuclear genes in the blue module, eight nuclear genes in the turquoise module, and seven nuclear genes in the yellow module were selected. To explore the functions of these nuclear genes, we used the potato database (http://solanaceae.plantbiology.msu.edu/index.shtml, accessed on 1 February 2024) and the NCBI database (https://www.ncbi.nlm.nih.gov, accessed on 1 February 2024) to query these nuclear genes’ functions. In addition, we annotated the functions of homologous genes in Arabidopsis using the TAIR database (https://www.arabidopsis.org, accessed on 1 February 2024) (Table 1). In the literature, we found that most of these nuclear genes are associated with root development and stress. For example, gene *AT3G03050* in *Arabidopsis thaliana*, which is homologous to *Soltu.DM.03G020780*, is involved in the plant cell wall and root hairs and plays a central role in plant growth, development, and stress response [26]. The gene *AT4G25480* in *Arabidopsis thaliana*, which is homologous to *Soltu.DM.03G016740*, belongs to the ERF/AP2 family of transcription factors and responds to stress and ABA [27]. The gene *AT1G27730* in *Arabidopsis thaliana*, which is homologous to *Soltu.DM.01G046560*, responds to salt stress and oxidative stress [28]. The *Arabidopsis thaliana* gene *AT1G70700*, which is homologous to *Soltu.DM.06G024860*, interacts with key transcription factors such as RHD6 and RSL1 to upregulate the expression of genes related to root hair development [29]. The gene *AT2G43010* in *Arabidopsis thaliana*, which is homologous to *Soltu.DM.07G014300*, balances plant growth and defense against adversity by encoding bHLH, a nuclear localization protein that interacts with active phyB proteins, among others [30].

#### 2.2.6. RT-qPCR (Real-Time Quantitative PCR) Validation of Core Genes in Candidate Modules in Gene Co-Expression Networks

**Figure 7:**
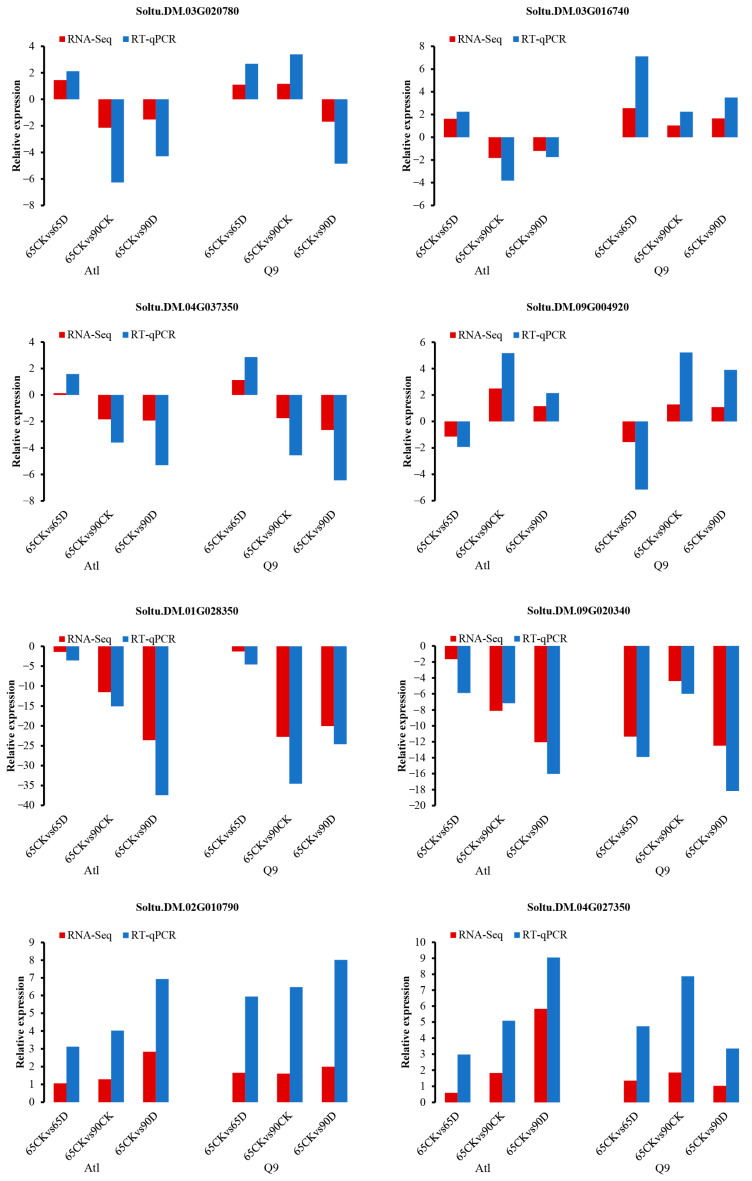

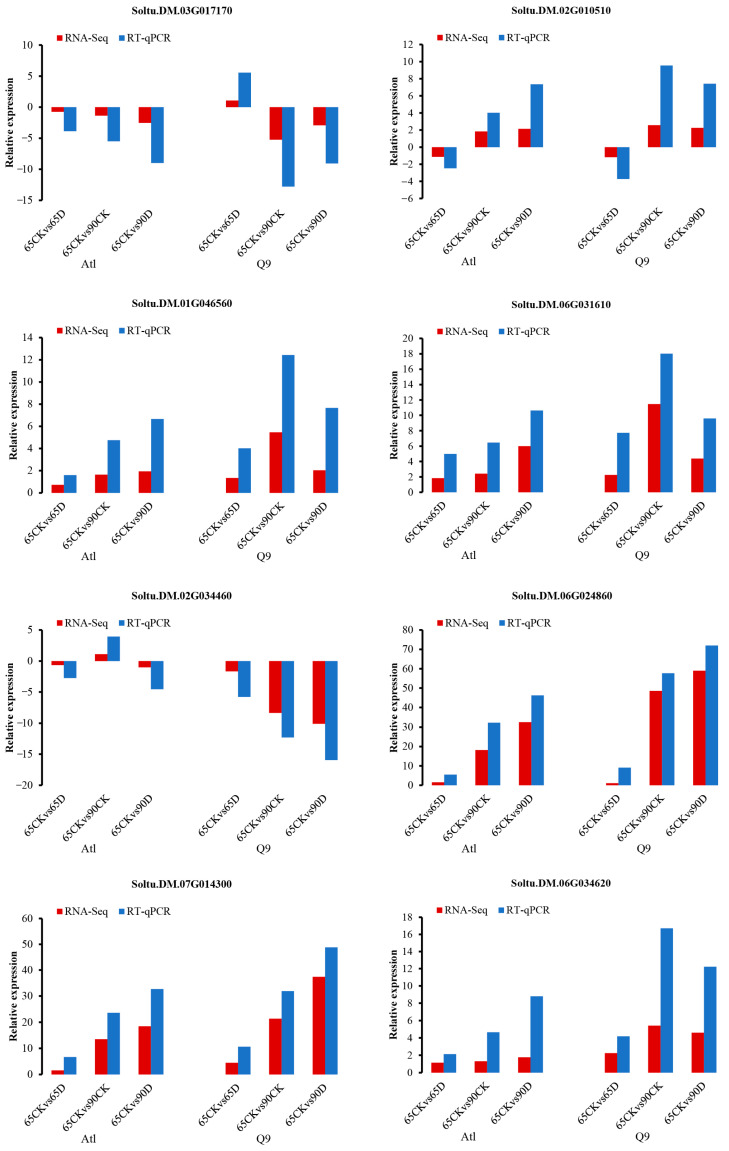
RT-qPCR Validation of Hub Genes.

Fifty-five core genes potentially related to the root response to drought stress were screened from the eight candidate gene modules selected from Atl and Q9 (Table 1). Among these genes are *Soltu.DM.03G020780*, *Soltu.DM.03G016740*, *Soltu.DM.04G037350*, *Soltu. DM.09G004920*, *Soltu.DM.01G028350*, *Soltu.DM.09G020340*, *Soltu.DM.02G010790*, *Soltu.DM.04G027350*, *Soltu.DM.03G017170*, *Soltu.DM.02G010510*, *Soltu.DM.01G046560*, *Soltu.DM.06G031610*, *Soltu.DM.02G034460*, *Soltu.DM.06G024860*, *Soltu.DM.07G014300*, and *Soltu.DM.06G034620* were selected for quantitative real-time fluorescence PCR analysis. The expression changes in these genes in different samples were analyzed, and the fluorescence quantification results were consistent with the transcriptome results (Figure 7, Supplementary Table S2). This is further evidence of the reliability of the transcriptome sequencing results. Among the genes tested, *Soltu.DM.06G024860* and *Soltu.DM.07G014300* showed the highest expression levels and were analyzed for subsequent validation of transgene function (Figure 7). 

### 2.3. Effects of Drought on Physiological and Biochemical Indices of Two Potato Genotypes

**Figure 8:**
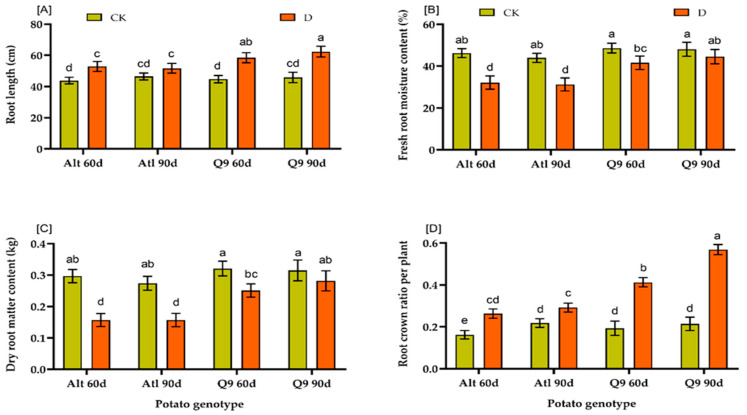
Effect of drought on the morpho-physiological indices of two potato genotypes: (**A**) Shows the effect on root length; (**B**) shows the effect on fresh root moisture content; (**C**) shows dry root matter content; (**D**) shows root crown ratio. Values represent the mean of 3 replicates; ±standard deviation (SD). Bars with different lowercase letters show significant differences by Duncan’s Multiple Range Test (*p* ≤ 0.05).

The measured morpho-physiological indices that showed significant differences in the present study included root length, fresh root matter content, dry root matter content, and root crown ratio (Figure 8A–D). The results showed that the two genotypes Atl and Q9 had a greater root length in droughted plants (Alt and Q9 60d, 90d plants) than the corresponding control plants (CK) after 90 days and 65 days under drought conditions. Genotype Q9 had the highest root length (62.38 cm) after 90 days, while Atl had the lowest root length (51.71 cm) after 90 days. For root moisture and dry root matter content, the control plants had the highest values after 60 and 90 days compared to the plants under drought stress in both genotypes but were significant in Atl. Comparing the two genotypes under drought stress, Q9 had the highest values after 90 days (Q9 90d) (44.56 and 0.282 kg), while Atl had the lowest values (31.26 and 0.157 kg). A similar result was obtained for the root crown ratio. The Q9 90d had the highest value (0.901), while the Atl 60d had the lowest value (0.415). 

**Figure 9:**
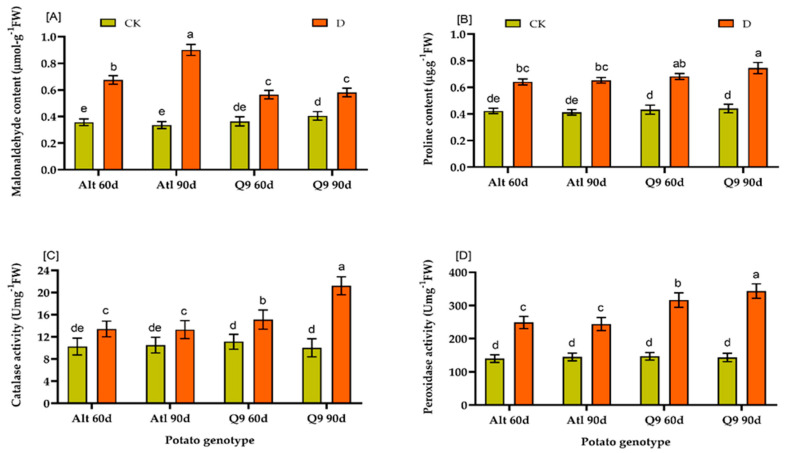
Effect of drought on the biochemical indices of two potato genotypes: (**A**) Shows the effect on MDA content; (**B**) shows the effect on proline content; (**C**) shows catalase activity; (**D**) shows peroxidase activity. Values represent the mean of 3 replicates; ± standard deviation (SD). Bars associated with different lowercase letters indicate significant differences by Duncan’s Multiple Range Test (*p* ≤ 0.05).

Biochemical indices such as MDA, proline, catalase and peroxidase showed significant differences between the treatment means. Genotype Atl had the highest value after 90 days (0.901), and genotype Q9 had the lowest value after 90 days (0.512). Both values were higher than those of the respective control plants. For proline content, Q9 had the highest value after 90 days (0.745), while Atl had the lowest value after 60 days (0.541). In the results for catalase and peroxidase, Q9 90d had the highest value (21.22 and 343.62), while Atl 90d had the lowest value (13.31 and 244.09) (Figure 9A–D).

## 3. Discussion

Traditional biological research focuses primarily on studying the effects of individual functional components such as DNA, mRNA, and proteins on life activities at the molecular level. Although this approach is important for uncovering the genetic mechanisms of certain traits, it can only provide localized explanations for the occurrence of certain life activities [31,32,33]. However, with the rapid development of sequencing technology, traditional biological research can no longer fully and effectively explore the biological meaning contained in large-scale data. In this context, bioinformatics has become an indispensable research tool in the field of systems biology, utilizing extensive data sources such as the genome, transcriptome, and metabolome [34]. Compared with other regulatory networks, WGCNA is a useful data analysis tool that can group genes into different modules based on their expression patterns in different samples, and these modules can be bioinformatically analyzed among themselves to determine their enrichment in a particular trait so that the modules containing genes that are most important for a trait can be predicted [35]. In addition, WGCNA can analyze similarities and differences between different samples and is also useful for comparative analyses. Therefore, WGCNA is widely used in numerous biological research fields, including genomics, proteomics, and metabolomics [36,37].

In this study, an enrichment analysis of eight tissue-specific modules was performed in two drought-tolerant cultivars, Atl and Q9, which have different levels of drought tolerance. The results showed that all eight modules could yield biologically significant regulatory pathways. In this study, we directly used the root tissues corresponding to the transcriptomic data, including roots, as the traits of interest under investigation. This approach has been widely reported in previous studies. For example, DiLeo et al. [38] investigated acyl sugar metabolism by comparing the transcriptomics of low and high-acyl sugar tomato cultivars using WGCNA (including six transcription factor genes and flavonoid metabolism genes) and plant defense-related genes (including genes putatively encoding NB-ARC and leucine-rich repeat sequences, protein kinases, and defense signaling proteins) [39]. To further elucidate the regulatory mechanism of fruit ripening, Kuang et al. [40] used a WGCNA-based enrichment method in combination with a time-stepped transcriptome analysis on banana fruit, leading to the construction of a regulatory network of transcription factors for ethylene-regulated ripening in banana fruit [41]. Li et al. [41] used the autotetraploid potato JC14 as an experimental subject to analyze the transcriptome of the root, stem, and leaf at the stages of seedling, tuber formation, and tuber expansion, uncovering thousands of DEGs and identifying 40 nodal genes related to carbohydrate metabolism, defense responses, and transcription factors using WGCNA. These results provide important insights for further understanding the molecular regulation and genetic mechanism of potato tissue development [42].

In this study, we focused on potato roots, which are a key organ in relation to water supply and drought. Although some progress has been made in analyzing the genetic mechanisms in root systems that serve as the basis for potato’s vital activities, such as the identification of numerous significant root resistance genes through quantitative trait locus and association analysis, these research approaches possess certain limitations. For example, quantitative trait loci localization cannot capture the genetic heterogeneity present in populations with complex genetic backgrounds. In addition, most agronomic traits are strongly correlated with the overall population structure. The WGCNA method, on the contrary, solves these problems by organizing thousands of genes with similar biological functions into coherent modules based on their co-expression patterns. By examining the biological significance of these modules, WGCNA enables a more comprehensive understanding of gene functions within a module. This approach provides better trait resolution in complex populations with diverse genetic backgrounds and is, therefore, widely applicable in high throughput genomic research [28,43,44,45].

The results of the enrichment analysis in this study show, for example, that among the enriched genes in the brown Atl module is the potato gene *Soltu.DM.03G020780*, which is a homolog of the *Arabidopsis thaliana* gene *AT3G03050*, which is involved in forming the plant cell wall and developing root hairs. These processes play a central role in plant growth, development, and response to stress [28]. Another gene in the brown module, *Soltu.DM.03G016740*, is the homolog of *AT4G25480* in *Arabidopsis thaliana* and belongs to the ERF/AP2 transcription factor family. It is known to respond to unfavorable stress conditions and ABA [29]. In the blue Q9 module, the homolog of the *Soltu.DM.01G046560* gene in *Arabidopsis thaliana*, which is also the homolog of *Soltu.DM.03G016740*, *AT1G27730*, can respond to salt and oxidative stress [46]. In the turquoise Q9 module, the homologous gene of *Soltu.DM.06G024860*, *AT1G70700* in *Arabidopsis thaliana*, interacts with key transcription factors such as RHD6 and RSL1 to upregulate the expression of genes related to root hair development [31]. In the yellow Q9 module, the *Soltu.DM.07G014300* gene homolog *AT2G014300* in *Arabidopsis thaliana* responds to salt and oxidative stress. Moreover, in the yellow Q9 module, the homolog of *AT2G43010*, *Soltu.DM.07G043010*, plays a crucial role in the balance between plant growth and defense against adversity. It encodes the nuclear localization protein bHLH, which interacts with active phyB proteins [32].

In addition, the Gene Ontology and KEGG enrichment analyzes revealed important pathways, especially in Q9. Thus, the analyses revealed a significant upregulation of DEGs and DEPs involved in the biosynthesis of secondary metabolites, including phenylpropanoids, terpenoids, and alkaloids. Zhao et al. [47] postulated that DEGs encoding key enzymes such as phenylalanine ammonialyase (PAL) and terpene synthases had increased expression, leading to increased production of stress-responsive compounds. These secondary metabolites play a crucial role in antioxidant defense, pathogen resistance, and stress signaling and contribute to the plant’s ability to cope with water scarcity [48]. Understanding the regulation of secondary metabolic pathways provides valuable insights into the molecular basis of drought tolerance in potatoes and helps in the development of resistant plant varieties for sustainable agriculture. 

It is known that plants use different ribosomal proteins for different purposes. For example, the versatile ribosomal protein S3 serves both as a structural and functional element of the ribosome and as a DNA base excision repair enzyme [49,50]. In our current study, the “ribosome” pathway was found to be one of the major KEGG pathways for DEGs in genetic information processing. The expression of DEGs and DEPs associated with ribosome biogenesis and function suggests possible adaptations of the protein synthesis machinery to water scarcity [51]. For example, the upregulation of ribosomal protein genes may increase the efficiency of protein synthesis under drought conditions and ensure the production of stress-responsive proteins. Additionally, changes in pyruvate metabolic pathways, including glycolysis and the TCA cycle, are observed with differential expression of key enzymes such as pyruvate kinase and pyruvate dehydrogenase [52]. The pyruvate metabolic pathway was significantly identified among the metabolic pathways in the KEEG enrichment analyzes. These metabolic adaptations contribute to regulating energy production and carbon flux to support important cellular processes and stress responses in potato roots under drought conditions.

Further analysis of DEGs and DEPs based on KEGG enrichment led to the identification of key pathways such as “zeatin biosynthesis”, which turns out to be a crucial aspect of plant adaptive mechanisms. Zeatin is a type of cytokinin hormone known for its role in promoting cell division, shoot growth, and stress response [53]. The analyses show the upregulation of genes involved in the biosynthesis of zeatin in potato roots under drought conditions, indicating a possible role of cytokinin signaling in drought tolerance mechanisms. Moreover, glycolysis is a metabolic pathway in which glucose is broken down for energy, while gluconeogenesis is the reverse process in which glucose is synthesized from noncarbohydrate sources [54]. Expression of DEGs and DEPs involved in glycolysis/gluconeogenesis pathways under drought conditions. For example, genes encoding key enzymes such as hexokinase, phosphofructokinase, and pyruvate kinase may exhibit altered expression levels to modulate energy production and carbohydrate metabolism in response to water scarcity [55]. In addition, analyzes of DEGs based on KEGG pathway enrichment show changes in the expression of DEGs and DEPs involved in the biosynthesis of “phytohormone signaling and starch and sucrose metabolism”. In the response of potato roots to drought, the expression of DEGs and DEPs associated with “phytohormone signaling” is of central importance. For example, the upregulation of DEGs, such as ABA-responsive element-binding factor (ABF) and PYR/PYL receptors underscores the activation of abscisic acid (ABA) signaling pathways that are critical for drought tolerance [56]. In addition, DEPs such as auxin-responsive proteins (IAAs) and gibberellin-regulated proteins (GAs) illustrate the intricate interaction between ABA and other phytohormones that orchestrate adaptive responses [57]. Comprehensive analyses reveal the nuanced interplay of DEGs and DEPs and provide insights into the sophisticated gene networks that control potato root resistance to drought stress. DEGs related to starch and sucrose metabolism are crucial in potato roots under drought stress [58]. In particular, DEGs such as starch synthase and sucrose phosphate synthase show upregulation, indicating increased starch and sucrose synthesis under stress conditions [59]. This increased expression underscores the plant’s adaptive strategy to accumulate energy reserves, which are critical for maintaining root growth in times of water scarcity.

In the present study, we also investigated the effects of drought on physiological and biochemical indices in the two potato genotypes Atl and Q9. We found that there was a significant difference between the two genotypes and the plants under drought conditions compared to the respective control plants after 60 and 90 days (Figure 8 and Figure 9). The plants under drought conditions formed longer roots compared to the control plants, and the same result was observed for the ratio of root crowns. At the same time, genotype Q9 formed longer roots and a greater root crown ratio than Atl. For fresh and dry root matter content, the control plants had significantly higher values than only the susceptible genotype Atl. These results are in line with research results that indeed show that plants exposed to drought often exhibit a phenomenon known as “root elongation” or “root proliferation”, in which they form longer roots compared to plants under well-watered conditions. This adaptive response enables plants to explore deeper soil layers in search of water and thus increase their chances of survival in times of water scarcity [60,61]. According to Kamanga et al. [62], high MDA accumulation is often associated with vulnerability to water scarcity and stress. The decreased activity of PS II during drought is associated with oxidative stress and cell membrane damage due to increased lipid peroxidation [63]. Our results showed that higher MDA content in drought-stressed plants was related to the effects of drought stress on them. MDA content increased significantly with increasing drought stress in the susceptible potato genotype Atl. Our result shows that drought stress promotes oxidative damage at all levels of the genotype, with the most severe damage occurring after 90 days. Proline also affects osmoregulation, membrane stability, detoxification, cytosolic pH adjustment, and maintenance of enzyme structure in cells [64]. In the present study, proline content was significantly higher in the drought-stressed plants than in the corresponding control plants, although there was no significant difference between the two genotypes. However, Q9 had a slightly higher proline content than Atl, the susceptible genotype. This result is in agreement with the findings of Per et al. [65], who observed that higher accumulation of proline was observed in tolerant plants under drought conditions. In addition, antioxidant enzymes CAT and POD were increased to a greater extent in the drought-tolerant genotype, Q9, after both 60 and 90 days, compared to the increases observed in the less drought tolerant genotype, Atl (Figure 9). Antioxidant enzymes are crucial for protecting plant cells from stress-induced cell damage caused by the formation of free radicals, especially ROS [66,67]. Therefore, it has been suggested that increasing the activity of antioxidant enzymes during stress could benefit plant growth and development.

These results, in general, suggest that genes with high connectivity to target genes in WGCNA have similar biological significance and may provide new research avenues for target gene searches. In this study, we mainly focused on analyzing eight highly related regulatory modules: brown, green, turquoise, and yellow in Atl, and brown, blue, turquoise, and yellow in Q9. Although the other gene modules were not discussed in detail, they may also contain enrichment pathways related to root resistance, and further investigation is needed to understand their biological significance. All trait-specific modules identified in this study contain genes related to root resistance and development. Therefore, the combination of co-expression networks and specific agronomic traits can be used to analyze the biological functions of the genes within the modules, identify core genes, and gain crucial insights into the analysis of the complex molecular regulatory mechanisms in plants. 

## 4. Materials and Methods

### 4.1. Plant Material

Two potato genotypes, Atlantic (Atl) (CIP 800827) and Qingshu 9 (Q9) (C92.140), were provided by the International Potato Research Center (Peru). These genotypes showed significant variations in root morphology and degree of drought resistance. The Key Laboratory of Crop Genetic Improvement and Germplasm Innovation of Gansu Agricultural College provided the original potato seeds of Atl and Q9.

### 4.2. Experimental Design

The potato plants were cultivated in pots with a volume of about 20 L. The pots had a diameter of 30 cm and a height of 40 cm. Gauze was attached to the inner wall of the pots, making it possible to easily remove the entire potato root system by removing the soil through the gauze. A mixture of nutrient soil and vermiculite in a 1:1 ratio was thoroughly mixed and poured into the pots. Seed potatoes of the same size, smooth surface, and free of bacteria were obtained from our tissue culture laboratory. Two plants were sown in each pot, leaving only one plant after emergence. All plants were watered normally until day 30, when a drought treatment was initiated. Half of the plants continued to be watered normally so that the soil moisture remained between 70% and 85% and served as the control group. The other half of the plants were subjected to a drought treatment, controlling the amount of irrigation and maintaining the soil moisture between 40% and 55% throughout the treatment process. The first sampling was performed on day 30, when the plants had reached 30 days of growth, and the stress treatment began. Subsequent sampling was carried out every 15 days until day 90. The water content in each treatment corresponded to the maximum water-holding capacity of the nutrient soil and vermiculite mixture. Three biological replicates were performed for each treatment. The seedlings’ roots were then collected, immediately frozen in liquid nitrogen, and stored at −80 °C. Half of each stored sample was used for RNA extraction and subsequent transcriptome and proteome sequencing. The other half was used for the determination of physiological and biochemical indices related to drought stress resistance.

### 4.3. Extraction and Detection of Total RNA and Proteins from the Roots

A total of 24 samples were collected under different drought stress conditions: two mother plants (Atl and Q9), one tissue (root), two treatments (CK and severe drought stress (D), two time points (60 and 90 days), and three replicates. These samples were used for transcriptome and proteome sequencing. The mRNA was extracted from total RNA using the Dynabeads mRNA DIRECT kit (Invitrogen, Waltham, MA, USA). The integrity was analyzed using 1.0% agarose gel electrophoresis. RNA was analyzed by sequencing the entire transcriptome. The protein extraction essentially proceeded as follows: First, lysis solution (1.5% SDS/100 mM Tris-Cl) was added to the sample, mixed well, and the tissue homogenate was centrifuged to obtain the supernatant; the acetone precipitation method was used to precipitate the proteins in the solution, then the complex solution (8 M urea/100 mM Tris-Cl) was added to the protein precipitate to dissolve it, then dithiothreitol was added and incubated at 37 °C. Iodoacetamide (IAA) was then added, and the alkylation reaction was conducted at room temperature in the dark to seal the sulfhydryl groups. The protein concentration was determined using the Bradford method. After protein quantification, 50 μg of the sample was collected for sodium dodecyl sulfate-polyacrylamide gel electrophoresis (SDS-PAGE), and the protein bands were observed after staining with caulophyllin blue. After reduction and alkylation, the sample was spiked with 100 mM Tris-HCl solution, and the urea concentration was diluted to less than 2 M. Trypsin was added to the sample at a 1:50 enzyme-to-protein ratio, and the sample was incubated overnight at 37 °C and shaken to digest the protein. The next day, trifluoroacetic acid (TFA) was added to complete digestion, and the supernatant was desalted with Sep Pak C18. The supernatant was dried and stored at −20 °C.

### 4.4. Construction and Sequencing of Whole Transcriptome Libraries and Mass Spectrometry of Proteome

The library was constructed using a Small RNA Sample Pre Kit (Lexogen, Vienna, Austria), which takes advantage of the unique structure of the 3′ and 5′ ends of small RNA (full phosphate group at the 5′ end and hydroxyl group at the 3′ end). To synthesize cDNA by reverse transcription, total RNA was used as the starting sample, and the joining sites were added directly to both ends of the small RNA. The cDNA was then synthesized and amplified using PCR. The target DNA fragments were then separated using PAGE electrophoresis and subjected to a cutting and recycling process to obtain the cDNA library. After library construction, Qubit 2.0 was used for preliminary quantification of DNA, and the library was diluted to 1 ng/uL. Subsequently, the insert size of the library was detected using a high-sensitivity Agilent 2100 (Santa Clara, CA, USA). After the insert size was determined to be as expected, the effective concentration of the library was accurately quantified using qPCR (the effective concentration of the library was >2 nM) to ensure the quality of the library. After passing the library check, different libraries were pooled according to the effective concentration and the targeted downstream data volume for Illumina SE50 sequencing (San Diego, CA, USA). Sequential sequencing with simultaneous synthesis was used. DNA polymerase, crossover primers, and four dNTPs with base-specific fluorescent markers (as in the Sanger sequencing method) were added simultaneously to the reaction system. The 3′-OH of these dNTPs is chemically protected so that only one dNTP can be added at a time. After the dNTPs were added to the synthesized strand, all unused free dNTPs and DNA polymerase were washed out. Next, the buffer needed for fluorescence excitation was added, the fluorescence signal was excited using a laser, and an optical device completed the recording of the fluorescence signal. Finally, computer analysis was used to convert the optical signal into sequencing bases. After recording the fluorescent signal, chemicals were added to quench the fluorescent signal and remove the dNTP-3’-OH protecting group so that the next round of sequencing could take place. Illumina sequencing technology, which adds only one dNTP at a time, solves the problem of accurately measuring the homopolymer lengths. 

For proteomic mass spectrometry data acquisition, a Q Exactive Plus mass spectrometer was used in conjunction with an EASY-nLC 1200 liquid spectrometry system. The peptide samples were dissolved in an up-sampling buffer, aspirated using an autosampler, and combined for separation on an analytical column (50 μm × 15 cm, C18, 2 μm, 100 A). Two mobile phases (mobile phase A: 0.1% formic acid and mobile phase B: 0.1% formic acid, 80% ACN) were used to create an analytical gradient. The flow rate of the liquid phase was set to 300 nL/min. Mass spectra were acquired in DIA mode with one MS1 scan (R = 70 K, AGC = 3 × 10^6^, Max IT = 30 ms, scan range = 350–1250 *m*/*z*) and 30 MS2 scans with variable windows (R = 17.5 K, AGC = 1 × 10^6^, Max IT = 50 ms) for each scan cycle. IT = 50 ms).

### 4.5. Quality Assessment of Sequencing Data

Raw data obtained from whole transcriptome sequencing contain spliced and low-quality reads. To ensure the quality of the information analysis, these raw data must be processed. Image data obtained from sequencing were converted into sequence data by base calling, in particular using the Illumina 1.8 + Phred + 33 algorithm. Raw reads usually have a length between 2 and 40. The raw reads were then subjected to indexing and quality control of base data. During this process, reads that contain spliced sequences were filtered out. If the N-content in a single-end sequencing read exceeded 10% of the number of bases in that read, these paired-end reads were removed. If a single-end sequencing read contained low-quality bases (Q Q ≤ 20) that make up more than 50% of the number of bases in this read, these paired reads are removed, as this information would interfere with the subsequent information analysis. In addition, the GC content distribution was examined to determine the presence of AT and GC segregation. Due to the random interruption of the sequence and the principle of double-stranded complementarity, the GC and AT contents in the sequenced reads should theoretically be the same at each position and remain essentially stable throughout the sequencing process. After filtering these raw data and checking the GC content distribution, the resulting high-quality clean reads are ready for further analysis.

### 4.6. Comparative Analysis of the Reference Sequences

For the comparative analysis, we used the potato genome “DM v6.1” and the corresponding annotation files as reference genomes. These reference files can be downloaded from the following website (http://spuddb.uga.edu/dm_v6_1_download. shtml, accessed on 15 February 2024). First, we used Hisat2 (version 2.2.10) to sequence the clean reads against the reference genome to obtain the positional information about the reference genome or gene, as well as the sequence feature information specific to the sequenced samples, and then we used the tool Samtools (version 0.1.19) to convert the SAM file into a BAM file, and we reordered the converted BAM file. The sequenced files were reorganized, and the sequenced files were used for transcript assembly using the software cufflinks (version 1.2), and the abundance of these transcripts was estimated [46,68,69].

### 4.7. Analysis of Differential Expression

The calculation of gene expression in the samples was performed with the HTSeq package in Python. The TPM value for each gene can be calculated based on the gene length and the number of fragments that align with the gene [70,71]. It is generally assumed that a gene is expressed if the absolute TPM value exceeds 1; otherwise, it is not expressed. The R package DESeq2 R (v3.5.2) was used to analyze the DEGs in the control and treatment groups, and replicate samples followed a negative binomial distribution model [72]. For error control, the Benjamini and Hochberg method was used to adjust *p*-values. DEGs with adjusted *p*-values of less than 0.01 were selected for further analysis in this experiment [73]. For proteome analysis, raw DIA data were analyzed using the DIA-NN software (v1.7.16) [74]. First, a spectral library was predicted using a deep learning algorithm in DIA-NN using the Uniprot database for *Solanum tuberosum* proteins. Protein quantification information was obtained by extracting raw DIA data from the predicted spectral library, and the spectral library was obtained using the MBR function. The final results were screened with an FDR of 1% for the parent ion and protein levels, and the ratio of the mean of all biological repeat quantification values for each protein was used as the FC of difference to screen proteins with an FC difference of 1.2 (2 for LFQ) or more and a *p*-value < 0.05 difference for subsequent analysis [75,76]. To investigate protein function, the identified proteins were annotated in functional databases, and the GO, KEGG, and COG databases were annotated with BLAST (blastp, evalue ≤ 1 × 10^−5^), and the results with the highest scores were selected as annotation results, and the structural domains were annotated using BLAST software (version 2.13.0) in conjunction with the Uniprot and InterPro databases. The structural domains were annotated using the BLAST software in conjunction with the Uniprot and InterPro databases [77]. Subcellular localization was predicted using MultiLoc2 [78].

### 4.8. Weighted Gene Correlation Network Analysis of Differential Genes in Atl and Q9

Weighted Gene Co-expression Network Analysis (WGCNA) is a novel and advanced method for analyzing the co-expression patterns of genes in multiple samples. The core idea is to group highly correlated genes in a module in a gene co-expression network and examine the correlation between these genes based on the strength of the connections between the modules and information regarding the amount of gene expression [79]. Using this analytical approach, genes with similar expression patterns can be clustered, allowing the assignment of gene clusters to phenotypic plant traits. The results of the gene cluster and association analysis led to the construction of regulatory networks. Compared with the traditional analysis mode that only focuses on DEGs, this advanced analysis method can divide the thousands of highly diverse genes into different gene sets based on the principle of clustering genes with similar expression patterns and then utilize the correlation analysis results between these core genes and phenotypic data, which not only fully utilizes the gene information obtained, but also correlates the genes with phenotypes, so that we can obtain a general idea regarding a specific gene’s functions. The genes that form the center of a regulatory network are usually referred to as core genes. They are key genes that regulate upstream and downstream gene expression, making these genes more worthy of in-depth study and analysis. To investigate and uncover further drought-resistant genes in potato root systems, we applied WGCNA and gene set-phenotype association analysis [79,80]. Therefore, we used the mRNAs corresponding to different genes and different proteins in Atl and Q9 root systems in response to drought stress as determined using the quadrant map analysis. In conjunction with the biochemical indices of root resistance and hormone data, we conducted an in-depth analysis to identify drought-resistant genes in the potato root systems.

#### 4.8.1. Checking for Outlier Samples

Since the results of the network module analysis can be biased by outlier samples, it is important to check whether outlier samples are present in the dataset before creating the network module to ensure the accuracy of the results. Outlier samples can be identified as those with a low correlation or as samples that cannot be effectively grouped in the dendrogram. In this study, the correlation coefficients for the expression levels of each sample in two vessels, Atl and Q9, were calculated and then plotted in a dendrogram (Supplementary Figure S2). The results showed that there was no significant difference between the three replicates for each sample within Atl (Supplementary Figure S2A) and Q9 (Supplementary Figure S2B), and each sample clustered in one group, indicating the absence of outlier samples in this study.

#### 4.8.2. Determination of Soft Thresholds in Gene Co-Expression Networks

To construct the co-expression network, the correlation coefficient between each gene needs to be calculated, and then a similarity matrix between the genes is obtained. The formula Smn = cor (xm, xn), S = [Smn], is used to calculate the similarity matrix, where Smn denotes the Pearson correlation coefficient between genes m and n, and S represents the similarity matrix. A weighted gene co-expression network was created using the WGCNA package in the R software program (version 4.3.3). To ensure that the network matched the scale-free network distribution, the “pick soft threshold” function in the WGCNA package was used to calculate the corresponding weighting values (Supplementary Figure S3). Based on the results, a soft threshold β = 10 was chosen for constructing the expression network in both the Atl and Q9 datasets.

#### 4.8.3. Number of Gene Clusters and Module Slices for Gene Co-Expression Networks

After determining the soft threshold β = 10, the similarity matrix is converted into an adjacency matrix using the formula Amn = [(1 + Smn)/2] × β, and then the adjacency matrix is converted into a topological overlap matrix (TOM). At the same time, the function Tom = 1 − TOM is used to obtain an inverse of the TOM to obtain a dissimilarity matrix. This step aims to eliminate the background noise and minimize the effects of pseudo-correlation. Finally, the dissimilarity matrix is subjected to hierarchical clustering using the blast function. The resulting clustering tree is then subjected to the dynamic tree-cut method, where genes with similar expression patterns are grouped in the same branch. Each branch corresponds to a co-expression module, with different colors representing different modules. The number of genes in a module is assigned a clustering correlation degree based on its transcripts per kilobase per million mapped reads (TPM) value, and genes with a higher clustering degree are assigned to the same module Supplementary (Figure S4).

### 4.9. GO Enrichment Analysis of DEGs and Proteins

GO is an internationally standardized classification system for the description of gene functions. It consists of three main parts: MFs, BPs, and Cellular Components [81]. MF is mainly used to describe the individual functions of genes and gene products, such as carbohydrate-binding or ATP hydrolase activity. In contrast, BP is used to describe the BPs in which the products encoded by the genes are involved, such as mitosis or purine metabolism. Cellular components are used to describe subcellular structure, location, and macromolecular complexes, such as nucleolus, telomeres, and complexes that recognize initiation. GO enrichment analysis is used to understand the functional expression of genes in different samples based on the gene distributions in GO. In this study, the R package clusterprofile [82,83] was used for GO enrichment analysis, and significantly enriched GO categories were identified based on a *p*-value threshold of less than 0.05.

### 4.10. KEGG Enrichment Analysis of DEGs and Proteins

In multicellular organisms, the collective biological functions of different proteins depend on their coordinated interactions, and the analysis of signaling pathways provides a deeper understanding of their biological roles. The KEGG (https://www.genome.jp/kegg, accessed on 20 February 2024) is a comprehensive data repository that summarizes information on genomes, biological pathways, diseases, drugs, and chemicals. By integrating genomic data with high-level functional information, KEGG provides a comprehensive resource for genome sequencing and other high-throughput experimental methods. KEGG is a comprehensive database that integrates information on genomes, biological pathways, diseases, drugs, and chemicals [84]. KEGG pathway analysis can be used to identify the most important biochemical metabolic and signaling pathways in which proteins are involved. The identified proteins were compared with the KEGG database using BLAST (blastp, evalue ≤ 1 × 10^−5^), and for the BLAST results of each sequence, the comparison result with the highest score was selected as the annotation result [85]. In this study, KEGG enrichment analysis was performed using the R package clusterprofile, and the statistical significance of the enriched KEGG pathways was determined by a *p*-value threshold of less than 0.05.

### 4.11. Determination of Morpho–Physiological Indices and Antioxidant Enzyme Activities, Proline, and MDA Content in the Roots

Root length (cm) was determined using a ruler by randomly selecting three plants from each replicate and taking the average of these data. The dry matter content of the roots was determined by drying in an oven at 105 °C for 24 h. The dry samples were weighed using an electronic balance and then the root crown ratio was calculated. The moisture content of the fresh roots (RMC) was determined according to the formula = fresh weight − dry weight/fresh weight × 100%.

Enzyme samples were prepared from frozen root tissue preserved at −80 °C. Evaluation of malonaldehyde (MDA) content Lipid peroxidation was measured by calculating the amount of MDA using the technique for thiobarbituric acid (TBA) presented in Hodges et al. [86]. Proline content was determined according to the method of Bates et al. [87]. The activities of catalase (CAT) and peroxidase (POD) in the root homogenate were determined using a reagent kit (Nanjing Jiancheng Bioengineering Institute, Nanjing, China) according to the manufacturer’s instructions.

## 5. Conclusions

In summary, the analysis of RNA-seq and DIA protein data obtained from two different potato cultivars with varying degrees of drought tolerance provides a valuable resource for the study of potato root biology, encompassing aspects such as root development and root response to drought-induced stress. By identifying transcriptomes and proteomes expressed at specific developmental stages and elucidating associated BPs, pathways, and co-expressed genes. This study sheds light on the intricate mechanisms underlying the potato root system. In addition, networks of co-expression genes that play key roles in different drought-tolerant potato root systems were identified, and important candidate genes for the specific response of the root system to drought stress were investigated. In particular, it was found that prolonged cell division and increased POD activity contribute significantly to deep rooting and drought tolerance observed in the potato cultivar “Q9”. Moreover, the genotype Q9 showed a certain degree of tolerance to drought stress in terms of physiological and biochemical indices. Under drought conditions, genotype Q9 formed longer roots and a higher root crown ratio compared to Atl. This adaptive response allows the plants to explore deeper soil layers in search of water, thus increasing their chances of survival in times of water scarcity. However, the MDA content increased significantly more with increasing drought stress in the susceptible potato genotype Atl than in Q9. The proline content, as well as CAT and POD activities, were higher in the Q9 genotype. In addition, most genes associated with the metabolic pathways identified in this study that regulate plants under drought stress, such as zeatin biosynthesis, glycolysis as a metabolic pathway, phytohormone signaling, and starch and sucrose metabolism, etc., were expressed in both genotypes, but they were more prominent in genotype Q9. Correlation analysis between phytochrome interacting factors (PIF) and Jasmonate-ZIM domain (JAZ) genes known to be related to rooting depth and drought stress response identified a total of 55 potential candidate genes related to drought stress. In general, this study demonstrates that integrating transcriptomic and proteomic association analyses, in combination with identifying co-expression networks and applying plant physiology approaches, can effectively identify candidate genes involved in root-specific responses to drought stress. The comprehensive findings presented here serve as a valuable resource to improve our understanding of how different drought-tolerant potato root systems respond to drought stress while providing important clues for the molecular breeding of drought-tolerant cultivars.

## Figures and Tables

**Table 1:** Functional annotations of core genes in different modules. #N/A represents the absence of homologous genes.

Varieties	Module	Hub Gene	Hub Gene	Gene Function
Atl	brown	*Soltu.DM.04G030030*	*AT4G08950*	Phosphate-responsive 1 family protein
Atl	brown	*Soltu.DM.03G016820*	*AT5G52060*	BCL-2-associated athanogene
Atl	brown	*Soltu.DM.03G020780*	*AT3G03050*	cellulose synthase-like D3
Atl	brown	*Soltu.DM.11G004580*	#N/A	Protein of unknown function (DUF1635)
Atl	brown	*Soltu.DM.03G016740*	*AT4G25480*	dehydration response element B1A
Atl	brown	*Soltu.DM.07G024430*	*AT5G53760*	Seven transmembrane MLO family protein
Atl	green	*Soltu.DM.04G037350*	*AT5G42220*	Ubiquitin-like superfamily protein
Atl	green	*Soltu.DM.06G031840*	*AT1G07400*	HSP20-like chaperones superfamily protein
Atl	green	*Soltu.DM.10G002820*	#N/A	phosphoenolpyruvate carboxylase
Atl	green	*Soltu.DM.12G010920*	#N/A	HSP20-like chaperones superfamily protein
Atl	green	*Soltu.DM.06G031830*	*AT1G18640*	3-phosphoserine phosphatase
Atl	green	*Soltu.DM.09G004920*	*AT5G03555*	permease, cytosine/purines, uracil, thiamine, allantoin family protein
Atl	turquoise	*Soltu.DM.01G028350*	*AT2G44380*	Cysteine/Histidine-rich C1 domain family protein
Atl	turquoise	*Soltu.DM.11G023040*	#N/A	plasma membrane intrinsic protein
Atl	turquoise	*Soltu.DM.10G019060*	#N/A	Peroxidase superfamily protein
Atl	turquoise	*Soltu.DM.01G051390*	*AT1G20330*	sterol methyltransferase
Atl	turquoise	*Soltu.DM.01G001830*	*AT1G50480*	10-formyltetrahydrofolate synthetase
Atl	turquoise	*Soltu.DM.03G033470*	*AT3G18080*	B-S glucosidase
Atl	turquoise	*Soltu.DM.06G021870*	*AT1G51630*	O-fucosyltransferase family protein
Atl	turquoise	*Soltu.DM.09G020340*	*AT3G22830*	heat shock transcription factor A6B
Atl	yellow	*Soltu.DM.04G029560*	*AT4G08850*	Leucine-rich repeat receptor-like protein kinase family protein
Atl	yellow	*Soltu.DM.02G010790*	*AT3G27170*	chloride channel B
Atl	yellow	*Soltu.DM.01G028700*	*AT2G44260*	Plant protein of unknown function (DUF946)
Atl	yellow	*Soltu.DM.04G027350*	*AT4G08850*	Leucine-rich repeat receptor-like protein kinase family protein
Atl	yellow	*Soltu.DM.04G029550*	*AT1G35710*	Protein kinase family protein with leucine-rich repeat domain
Atl	yellow	*Soltu.DM.03G037250*	*AT1G53903*	Protein of unknown function (DUF581)
Atl	yellow	*Soltu.DM.04G029480*	*AT4G08850*	Leucine-rich repeat receptor-like protein kinase family protein
Q9	brown	*Soltu.DM.02G006090*	*AT5G25610*	BURP domain-containing protein
Q9	brown	*Soltu.DM.01G003450*	*AT1G70520*	cysteine-rich RLK (RECEPTOR-like protein kinase)
Q9	brown	*Soltu.DM.10G022490*	#N/A	PYR1-like
Q9	brown	*Soltu.DM.03G017170*	*AT5G52190*	Sugar isomerase (SIS) family protein
Q9	brown	*Soltu.DM.02G010510*	*AT5G40510*	Sucrase/ferredoxin-like family protein
Q9	brown	*Soltu.DM.07G027920*	*AT1G55690*	Sec14p-like phosphatidylinositol transfer family protein
Q9	brown	*Soltu.DM.01G042680*	*AT1G71900*	Protein of unknown function (DUF803)
Q9	blue	*Soltu.DM.01G046560*	*AT1G27730*	salt tolerance zinc finger
Q9	blue	*Soltu.DM.06G031610*	*AT5G59480*	Haloacid dehalogenase-like hydrolase (HAD) superfamily protein
Q9	blue	*Soltu.DM.03G024660*	*AT3G48520*	cytochrome P450, family 94, subfamily B, polypeptide
Q9	blue	*Soltu.DM.03G028100*	*AT1G60190*	ARM repeat superfamily protein
Q9	blue	*Soltu.DM.03G024690*	*AT3G48520*	cytochrome P450, family 94, subfamily B, polypeptide
Q9	blue	*Soltu.DM.03G002340*	*AT1G61260*	Protein of unknown function (DUF761)
Q9	turquoise	*Soltu.DM.06G017960*	*AT2G35710*	Nucleotide-diphospho-sugar transferases superfamily protein
Q9	turquoise	*Soltu.DM.01G029490*	*AT1G05160*	ent-kaurenoic acid hydroxylase
Q9	turquoise	*Soltu.DM.02G034460*	*AT3G02040*	senescence-related gene
Q9	turquoise	*Soltu.DM.06G024860*	*AT1G70700*	jasmonate-zim-domain protein
Q9	turquoise	*Soltu.DM.06G026270*	*AT1G74790*	catalytics
Q9	turquoise	*Soltu.DM.04G012230*	*AT1G76690*	12-oxophytodienoate reductase
Q9	turquoise	*Soltu.DM.10G019340*	#N/A	PYR1-like
Q9	turquoise	*Soltu.DM.07G013340*	#N/A	Bax inhibitor-1 family protein
Q9	yellow	*Soltu.DM.07G014300*	*AT2G43010*	phytochrome interacting factor 3-like
Q9	yellow	*Soltu.DM.11G004020*	#N/A	Plant stearoyl-acyl-carrier-protein desaturase family protein
Q9	yellow	*Soltu.DM.04G020610*	*AT3G48310*	cytochrome P450, family 71, subfamily A, polypeptide
Q9	yellow	*Soltu.DM.02G027580*	*AT2G22910*	N-acetyl-l-glutamate synthase
Q9	yellow	*Soltu.DM.08G020150*	*AT4G19230*	cytochrome P450, family 707, subfamily A, polypeptide
Q9	yellow	*Soltu.DM.06G034620*	*AT3G22600*	Bifunctional inhibitor/lipid-transfer protein/seed storage 2S albumin superfamily protein
Q9	yellow	*Soltu.DM.09G025790*	*AT1G06760*	winged-helix DNA-binding transcription factor family protein

## Data Availability

The original contributions presented in the study are publicly available. This data can be found here: National Center for Biotechnology Information (NCBI) BioProject database under accession number PRJNA1032423 and iProX database under accession number PXD046520.

## Supplementary Materials

The following supporting information can be downloaded at https://www.mdpi.com/article/10.3390/plants13111530/s1, Supplementary Figure S1: Correlation analysis of whole transcriptome data (A) and proteome data (B) from potato Atl and Q9 roots under drought stress; Supplementary Figure S2: Cluster trees for all samples in Atl (A) and Q9 (B); Supplementary Figure S3: Determination of soft thresholds for gene co-expression networks in Atl (A) and Q9 (B); Supplementary Figure S4: Gene clustering tree and module sections for gene co-expression networks in Atl (A) and Q9 (B). The color representation module, each module with different colors, corresponds to a branch in the gene clustering tree; Supplementary Figure S5. Association analysis of gene co-expression network modules with physiological and biochemical traits in Atl (A) and Q9 (B). The horizontal axis represents different characteristics, and the vertical axis represents the eigenvectors of individual modules. The red grid plots have a positive correlation with the module, while the green grid plots have a negative correlation with the module; Supplementary Figure S6. Correlation of MEs between different modules of the gene co-expression networks in Atl (A) and Q9 (B); Supplementary Figure S7: The expression levels of all genes and the corresponding MEs in the different modules for each sample. The above figure shows the expression level of all genes in each sample in a module (Atl: A: brown module, B: green module, C: turquoise module, D: yellow module; Q9: E: brown module, F: blue module, G: yellow module, H: turquoise module). The rows represent the module genes, and the columns represent the samples. The following figure shows the ME expression level in a module in the corresponding sample; Supplementary Table S1: Statistics on the quality of whole transcriptome sequencing data outputs; Supplementary Table S2: Primer for qPCR; Supplementary Figure S8: GO enrichment of genes in Atl (A–D) and Q9 (E–H) candidate modules.
