# Supplementary material for: Comprehensive Transcriptome and Proteome Analyses Reveal the Drought Responsive Gene Network in Potato Roots
Source: Plants (Basel). 2024 May 31;13(11):1530. doi: 10.3390/plants13111530 (PMC11175002; doi:10.3390/plants13111530)
Supplement: Supplementary file 1 [file plants-13-01530-s001.zip › plants-2977398-supplementary.pdf]

Supplementary data

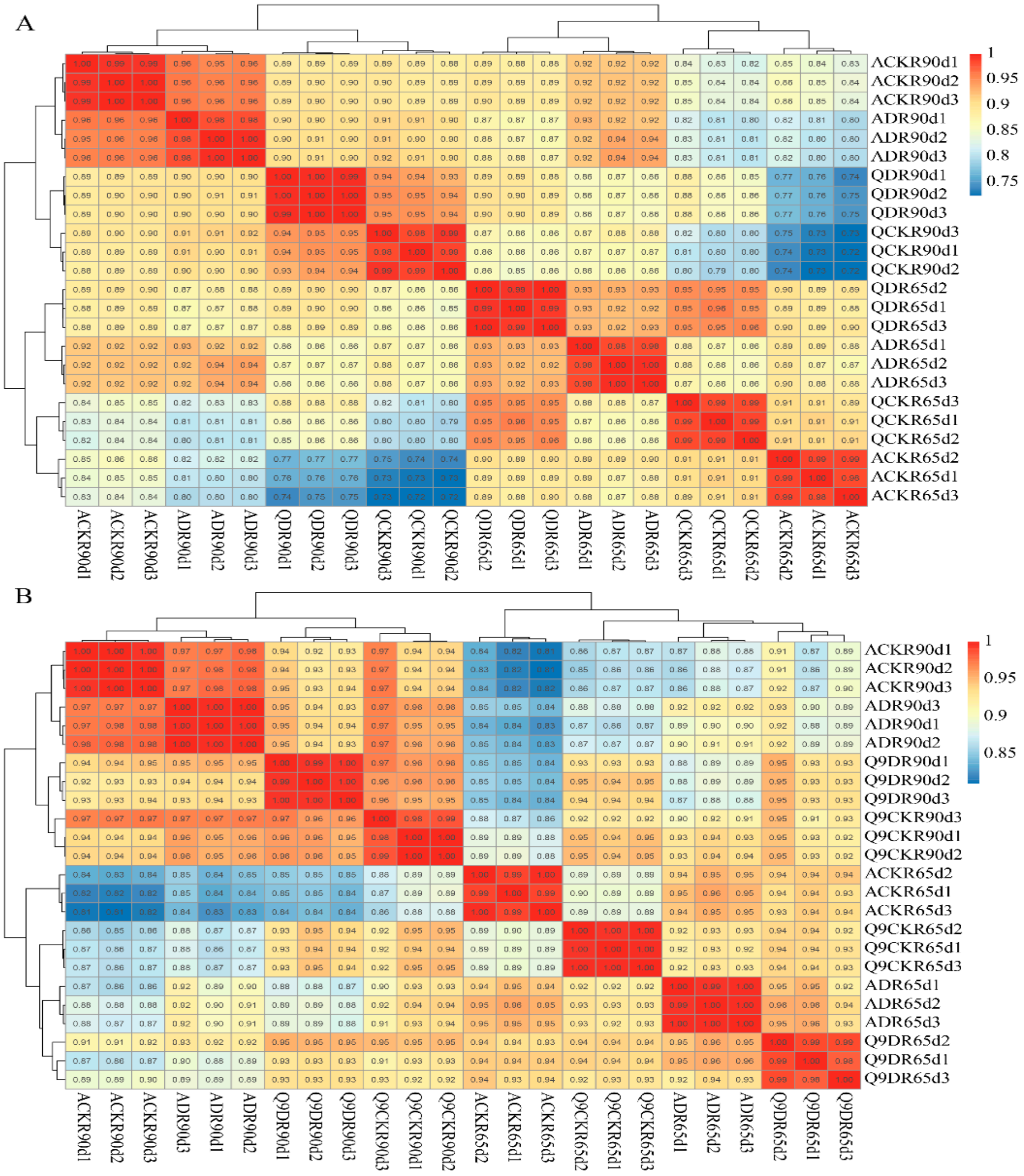

Supplementary Figure S1. Correlation analysis of whole transcriptome data (A) and proteome data (B) from potato Atl and Q9 roots under drought stress.

Supplementary Table S1. Statistics on the quality of whole transcriptome sequencing data outputs

Sample	raw reads	Q30	GC content	clean reads
ACKR65d1	13195926	96.50%	56.14%	12970619 (98.29%)
ACKR65d2	12368029	96.23%	54.49%	12082540 (97.69%)
ACKR65d3	15488308	97.06%	53.67%	14502617 (93.64%)
ACKR90d1	11211548	96.77%	53.39%	10282401 (91.71%)
ACKR90d2	12837096	96.82%	53.94%	12705840 (98.98%)
ACKR90d3	12475721	95.86%	53.55%	11744637 (94.14%)
ADR65d1	14209315	97.19%	54.47%	13553359 (95.38%)
ADR65d2	11602121	97.05%	53.92%	10756226 (92.71%)
ADR65d3	10938681	95.56%	54.44%	10720775 (98.01%)
ADR90d1	11450610	96.71%	53.40%	10414095 (90.95%)
ADR90d2	11459592	97.23%	54.20%	10850527 (94.69%)
ADR90d3	11740528	97.06%	53.55%	11017736 (93.84%)
QCKR65d1	12858739	96.83%	54.03%	12134701 (94.37%)
QCKR65d2	12027452	95.48%	53.02%	11070250 (92.04%)
QCKR65d3	10832198	96.78%	53.08%	10144278 (93.65%)
QCKR90d1	14428139	96.86%	51.66%	13799160 (95.64%)
QCKR90d2	15083062	97.37%	52.80%	14861935 (98.53%)
QCKR90d3	15520637	98.23%	53.58%	15256714 (98.30%)
QDR65d1	13840936	97.13%	54.09%	13043044 (94.24%)
QDR65d2	13599481	97.19%	54.09%	12691833 (93.33%)
QDR65d3	10564937	96.07%	53.44%	10379616 (98.25%)
QDR90d1	11760507	95.72%	52.14%	10696295 (90.95%)
QDR90d2	12851607	96.00%	52.23%	11809853 (91.89%)
QDR90d3	12670640	95.65%	52.42%	11846391 (93.49%)

Supplementary Table S2. Primer for qPCR

Gene name	Primer (5'– 3')
Soltu.DM.01G028350-F	TTGCAATGCTTGTGGGTCTG
Soltu.DM.01G028350-R	AGCTCCAATTGATGCGTGTG
Soltu.DM.01G046560-F	AACCGGTTCGATTGGTGTTG
Soltu.DM.01G046560-R	TTATGCCCACCAAGTGCTTG
Soltu.DM.02G010510-F	TGTGGCTGCTTGTTCACATG
Soltu.DM.02G010510-R	TGCCCAGCAATTTTCCCTTC
Soltu.DM.02G010790-F	TTTGGGATTGCTGTGCCATC
Soltu.DM.02G010790-R	AAGCAAGCGACCATAAGC
Soltu.DM.02G034460-F	ATTTGTTGCAGTCCGCTGAC
Soltu.DM.02G034460-R	GGCAATCATCCTTTGTCACC
Soltu.DM.03G016740-F	TCATGACGTGGCAGCTTTAG
Soltu.DM.03G016740-R	AACCTCCAAGCAGAATCAGC
Soltu.DM.03G017170-F	ACGATGGCGGATGATAATGC
Soltu.DM.03G017170-R	AGCACAAACATCGCTCCTTC
Soltu.DM.03G020780-F	TCAATTGCCCAAGCTTTGCC
Soltu.DM.03G020780-R	TGGATCAGCGGTAGAGACAA
Soltu.DM.04G027350-F	TCATTTTCGCCAGCATCAGG
Soltu.DM.04G027350-R	TGATGAAGACACGCGAATGC
Soltu.DM.04G037350-F	AGATCCACCTGCAGTACAGT
Soltu.DM.04G037350-R	TGCTGCAATGACTTGAGCAC
Soltu.DM.06G024860-F	TTGCATCAGCTGGATTGGTG
Soltu.DM.06G024860-R	TTGCGTGTGGGAACATGATG
Soltu.DM.06G031610-F	TGCAAATGAAGCCCATGTGG
Soltu.DM.06G031610-R	TTGTACACTGCCGTTGTTGG
Soltu.DM.06G034620-F	ATCCCCAATGCCTTTGTGTG
Soltu.DM.06G034620-R	ACCTGTTGGTCCATTTGCAG
Soltu.DM.07G014300-F	ATCCAATGTGCAGCTTGGTG
Soltu.DM.07G014300-R	ATTGCTTCCACAGTGGCTTG
Soltu.DM.09G004920-F	ACCAACGTCGATCGATCAAC
Soltu.DM.09G004920-R	TATGAGGGCACACCAACAAC
Soltu.DM.09G020340-F	TCGGTCCTTGTGTCGAATTG
Soltu.DM.09G020340-R	TTGCTTGTCACGCCTCAATC
actin-F	AGGAGCATCCTGTCCTCCTAA
actin-R	CACCATCACCAGAGTCCAACA

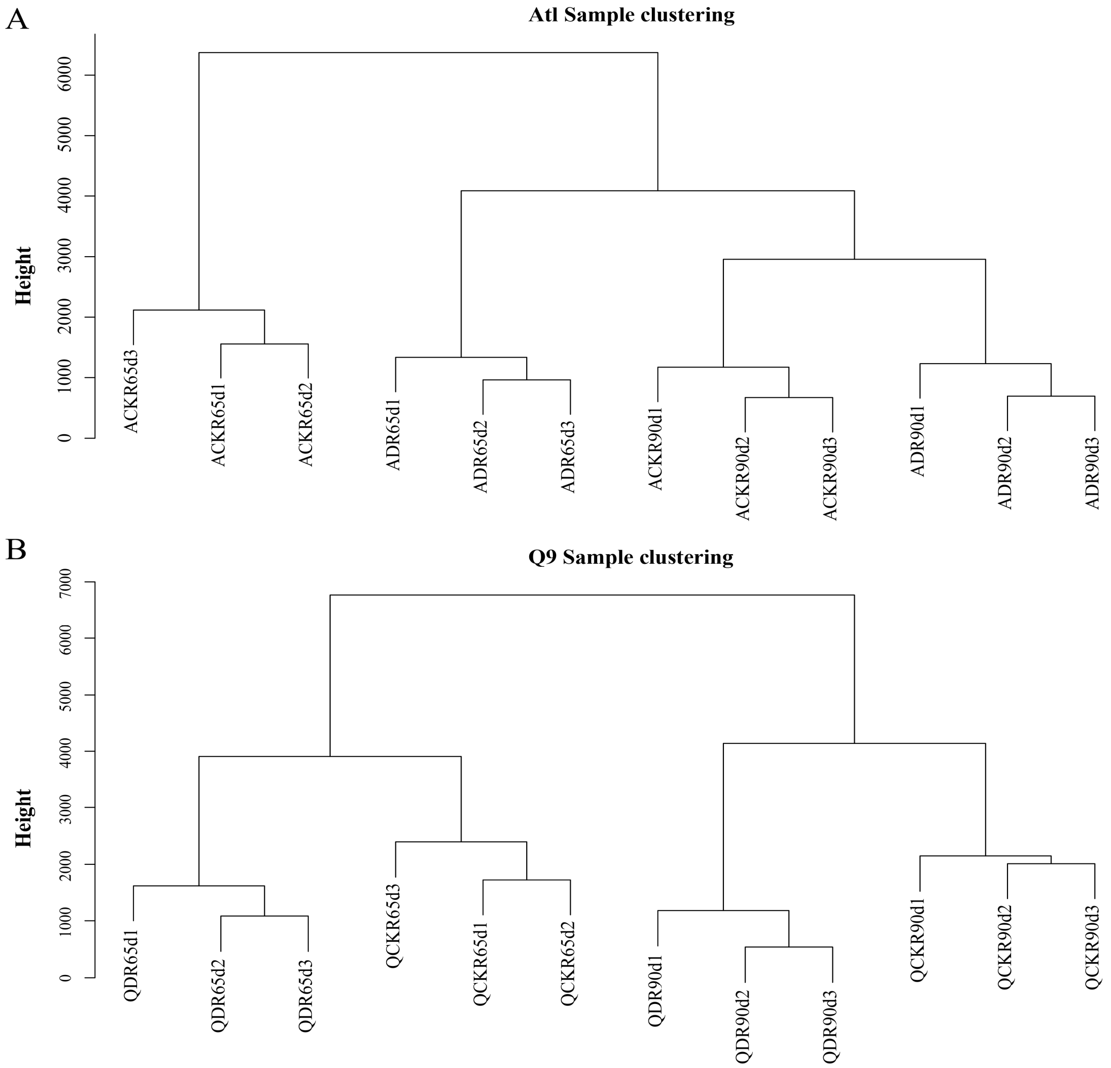

Supplementary Figure S2. Cluster trees for all samples in Atl (A) and Q9 (B)

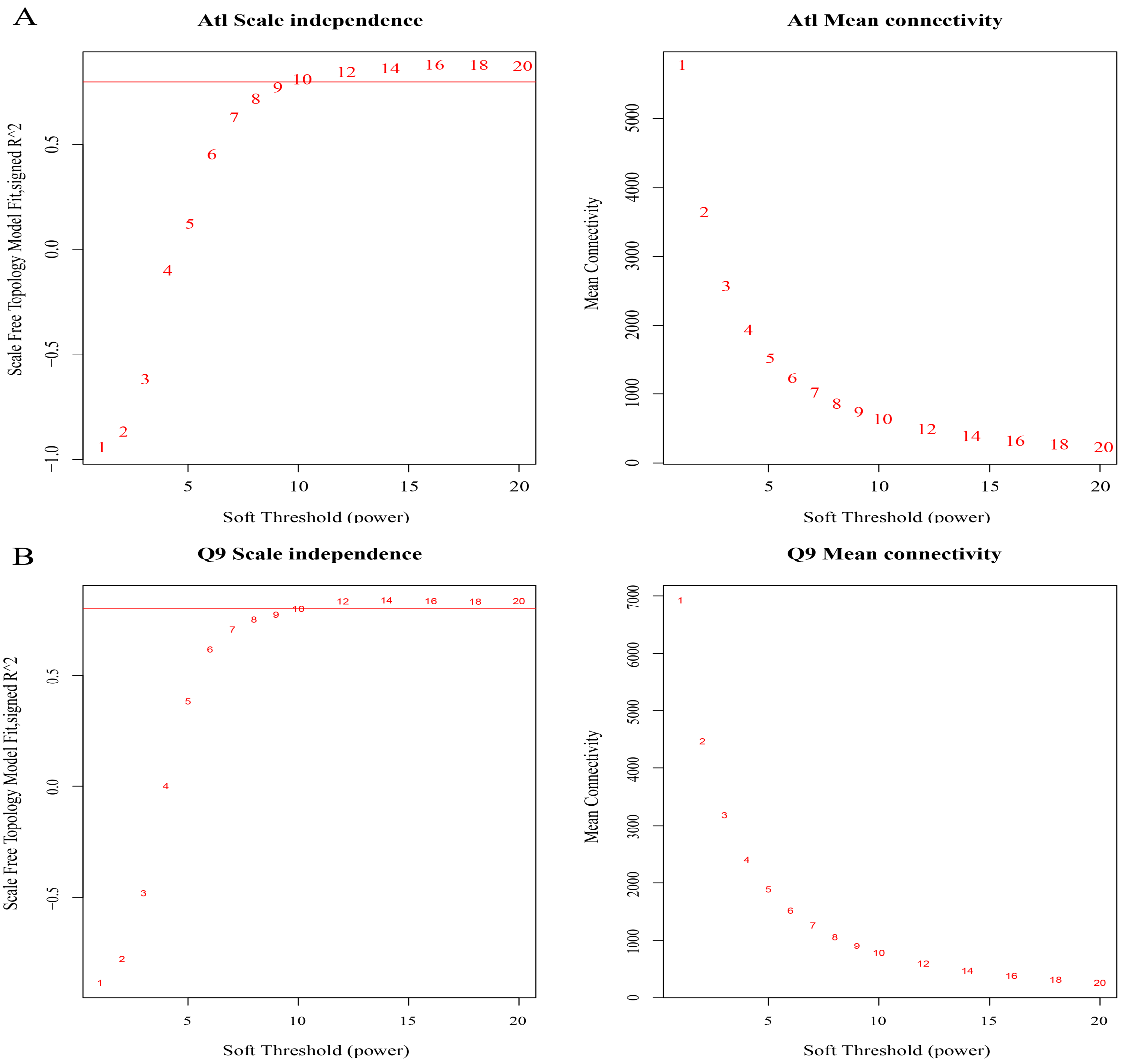

Supplementary Figure S3. Determination of soft thresholds for gene co-expression networks in Atl (A) and Q9 (B)

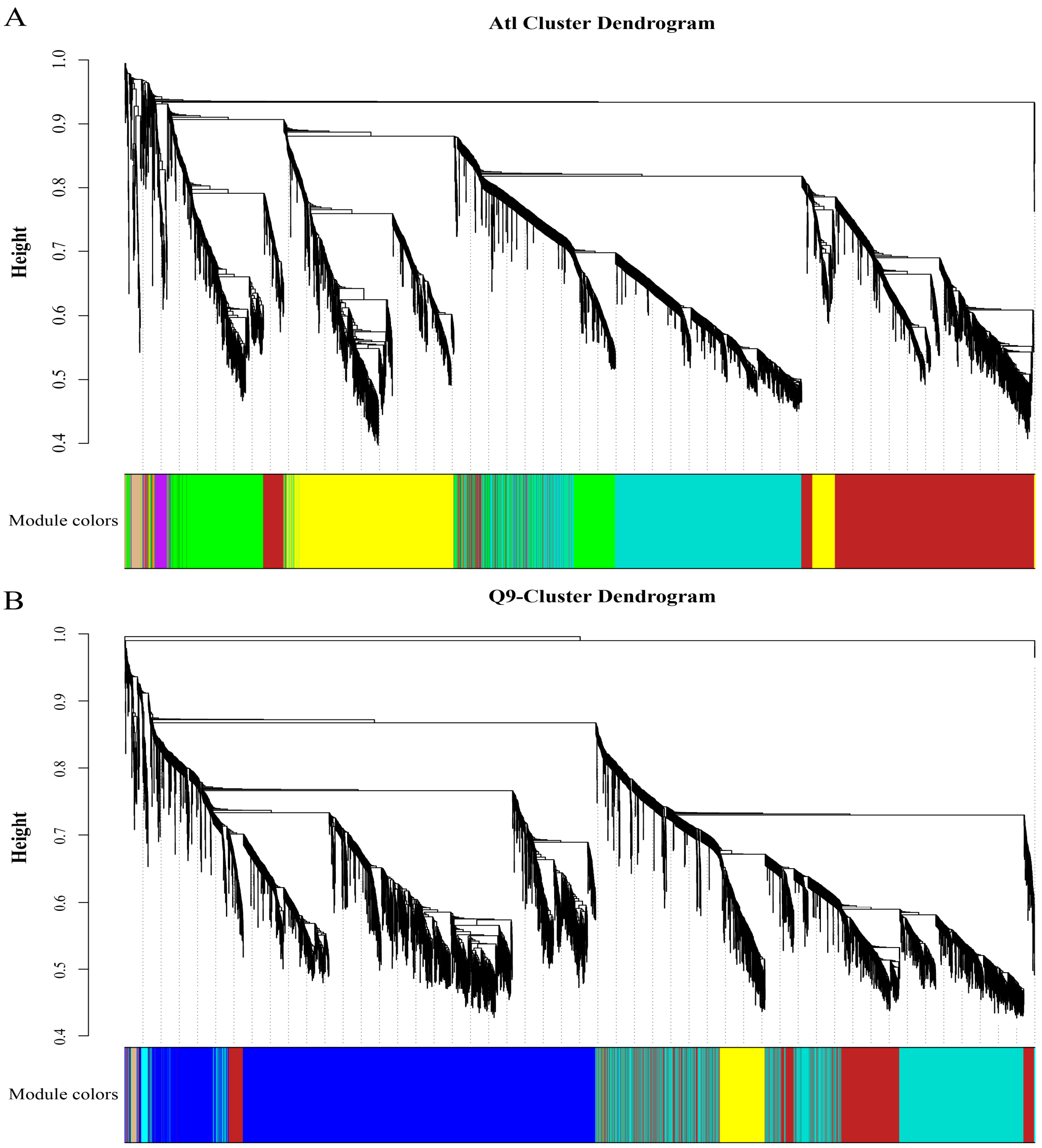

Supplementary Figure S4. Gene clustering tree and module sections for gene co-expression networks in Atl (A) and Q9 (B). The color representation module, each module with different colors, corresponds to a branch in the gene clustering tree.

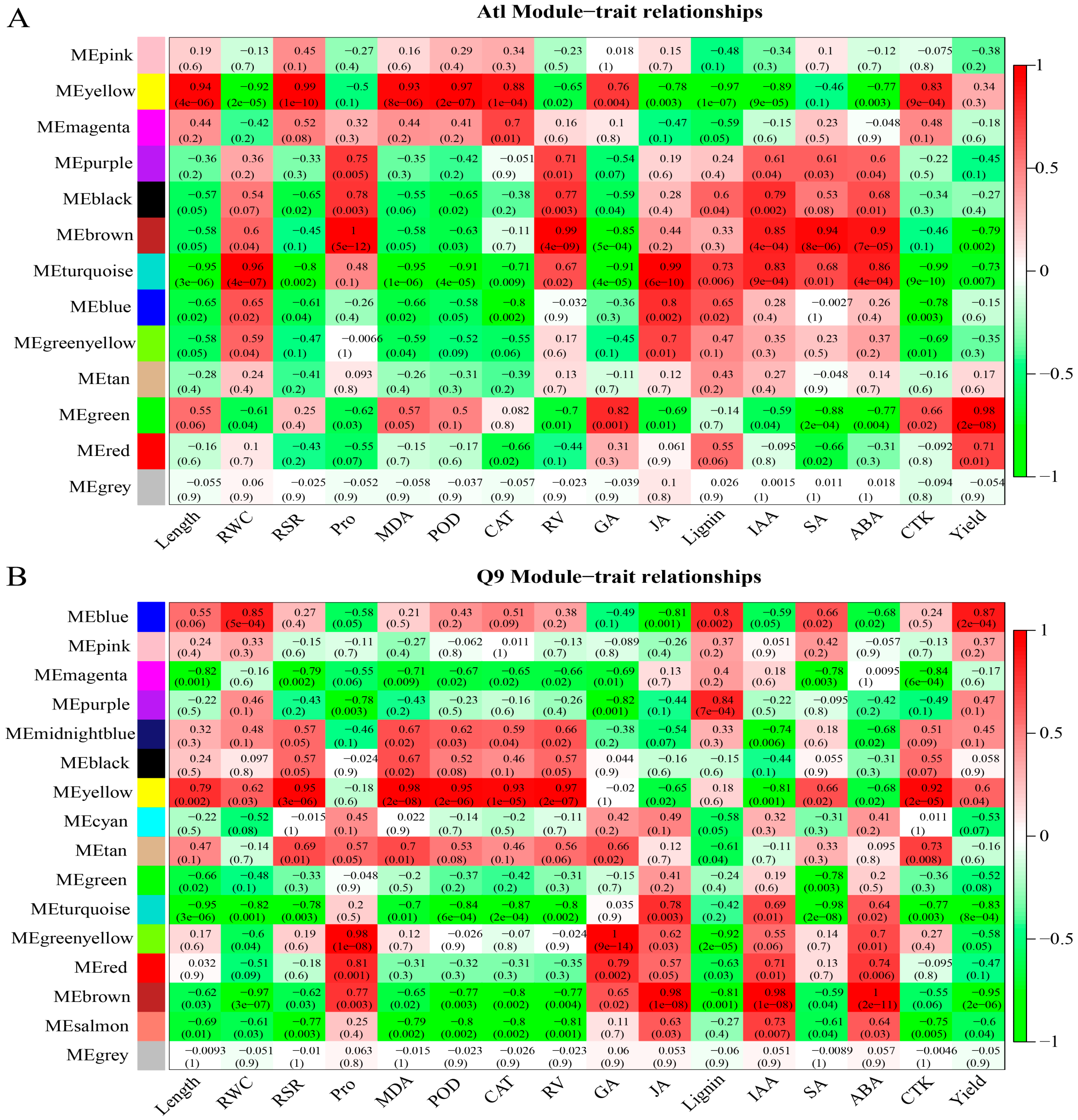

Supplementary Figure S5. Association analysis of gene co-expression network modules with physiological and biochemical traits in Atl (A) and Q9 (B). The horizontal axis represents different characteristics, and the vertical axis represents the eigenvectors of individual modules. The red grid plots have a positive correlation with the module, while the green grid plots have a negative correlation with the module.

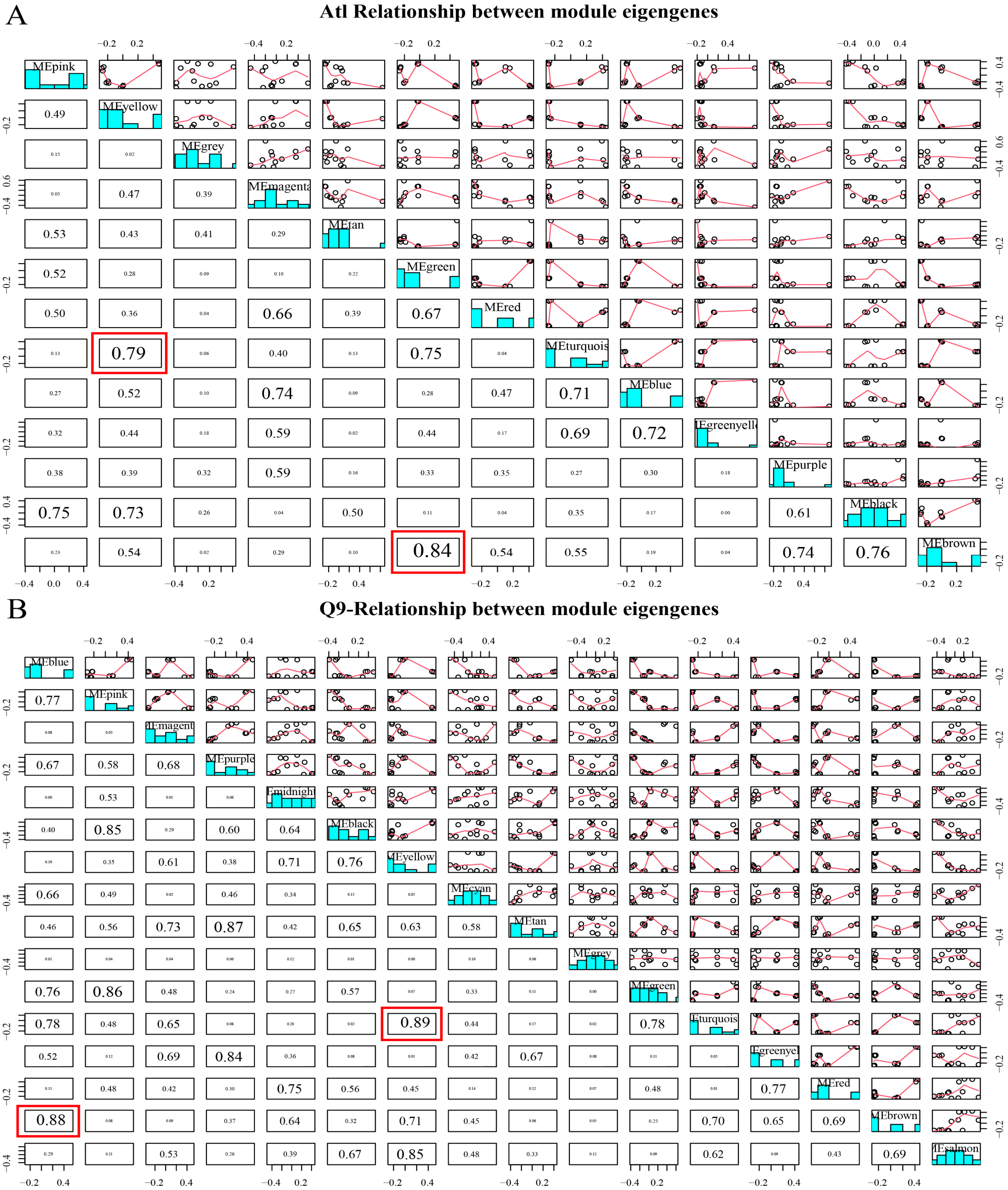

Supplementary Figure S6. Correlation of MEs between different modules of the gene co-expression networks in Atl (A) and Q9 (B)

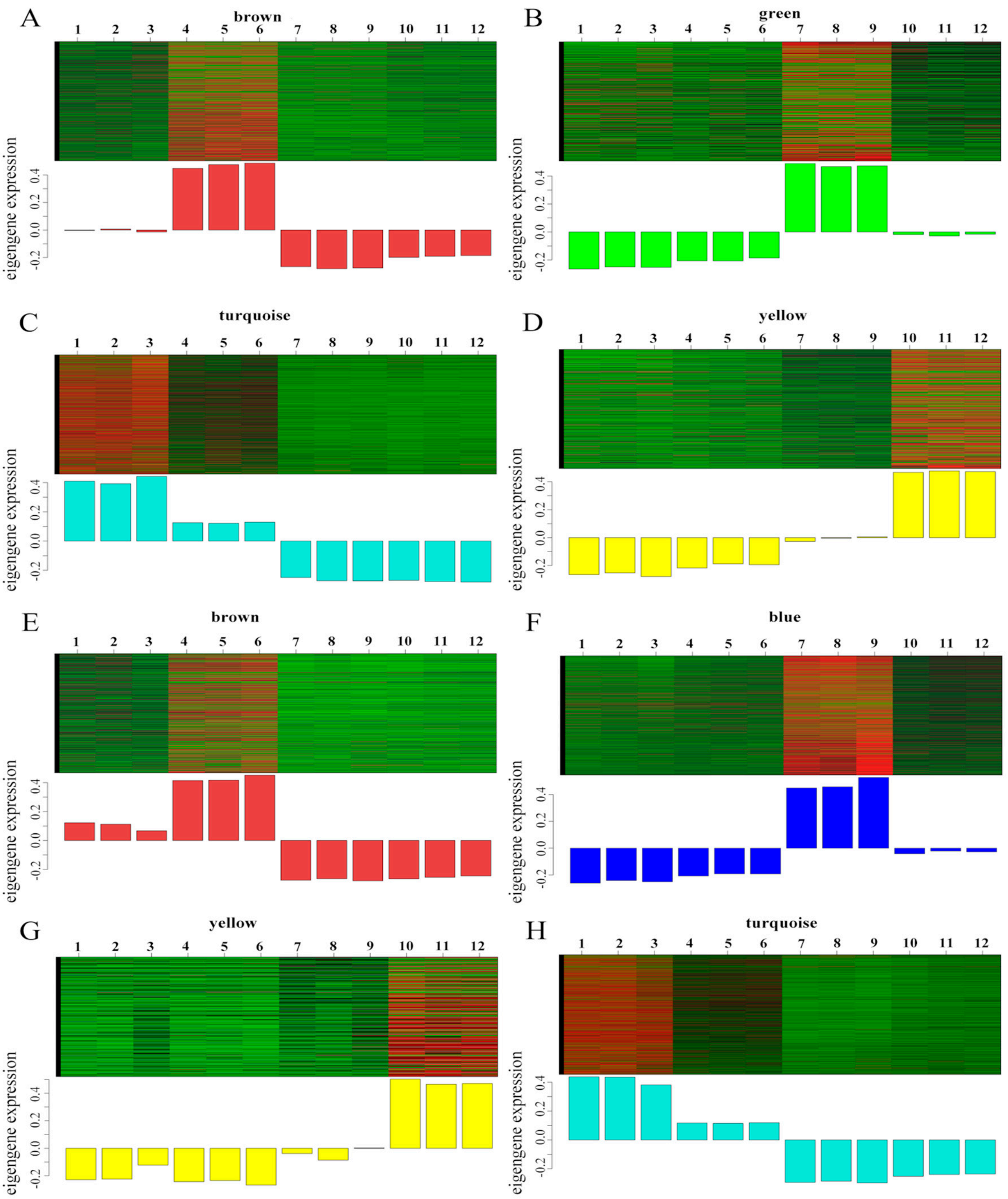

Supplementary Figure S7. **The expression levels of all genes and the corresponding MEs in the different modules for each sample.** The above figure shows the expression level of all genes in each sample in a module (Atl: A: brown module, B: green module, C: turquoise module, D: yellow module; Q9: E: brown module, F: blue module, G: yellow module, H: turquoise module). The rows represent the module genes, and the columns represent the samples. The following figure shows the ME expression level in a module in the corresponding sample.

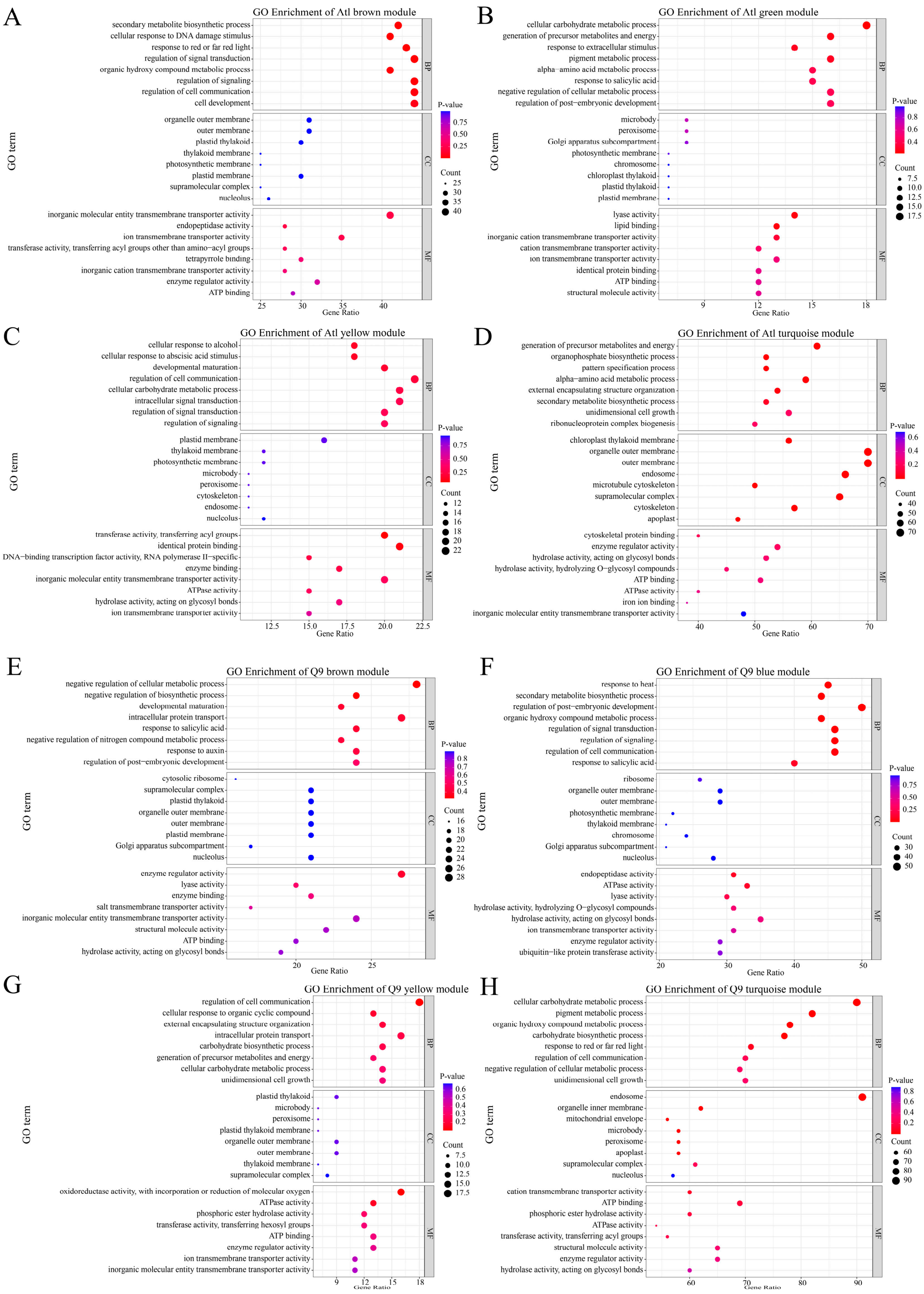

Supplementary Figure S8: GO enrichment of genes in Atl (A-D) and Q9 (E-H) candidate modules.
